# Accurate Prediction of Secreted Substrates and Identification of a Conserved Putative Secretion Signal for Type III Secretion Systems

**DOI:** 10.1371/journal.ppat.1000375

**Published:** 2009-04-24

**Authors:** Ram Samudrala, Fred Heffron, Jason E. McDermott

**Affiliations:** 1 Department of Microbiology, University of Washington, Seattle, Washington, United States of America; 2 Department of Molecular Microbiology and Immunology, Oregon Health and Science University, Portland, Oregon, United States of America; 3 Computational Biology and Bioinformatics, Pacific Northwest National Laboratory, Richland, Washington, United States of America; The Rockefeller University, United States of America

## Abstract

The type III secretion system is an essential component for virulence in many Gram-negative bacteria. Though components of the secretion system apparatus are conserved, its substrates—effector proteins—are not. We have used a novel computational approach to confidently identify new secreted effectors by integrating protein sequence-based features, including evolutionary measures such as the pattern of homologs in a range of other organisms, G+C content, amino acid composition, and the N-terminal 30 residues of the protein sequence. The method was trained on known effectors from the plant pathogen *Pseudomonas syringae* and validated on a set of effectors from the animal pathogen *Salmonella enterica* serovar Typhimurium (*S.* Typhimurium) after eliminating effectors with detectable sequence similarity. We show that this approach can predict known secreted effectors with high specificity and sensitivity. Furthermore, by considering a large set of effectors from multiple organisms, we computationally identify a common putative secretion signal in the N-terminal 20 residues of secreted effectors. This signal can be used to discriminate 46 out of 68 total known effectors from both organisms, suggesting that it is a real, shared signal applicable to many type III secreted effectors. We use the method to make novel predictions of secreted effectors in *S.* Typhimurium, some of which have been experimentally validated. We also apply the method to predict secreted effectors in the genetically intractable human pathogen *Chlamydia trachomatis*, identifying the majority of known secreted proteins in addition to providing a number of novel predictions. This approach provides a new way to identify secreted effectors in a broad range of pathogenic bacteria for further experimental characterization and provides insight into the nature of the type III secretion signal.

## Author Summary

Pathogenic bacteria release a number of different proteins that function to interfere with host defenses and allow bacterial invasion, persistence, and replication in the host. In many bacterial pathogens, the type III secretion system is used to inject these virulence factors directly to the cytoplasm of the host cell. The secreted proteins do not have well-conserved sequences and do not have any kind of common identifiable signal sequence to target them for secretion. This makes it very difficult to identify secreted proteins of this kind without experimental investigation, as can be done in other secretion systems. In this study, we develop a computational approach to detect secreted virulence factors from genomic protein sequences. We use this method to compare the N-terminal regions of proteins from *S.* Typhimurium and a plant pathogen, *P. syringae*, and show that this approach is the most effective method of computational identification of type III secreted proteins to date. We further use this approach to identify a sequence pattern in these proteins that presumably helps direct virulence proteins to the type III secretion apparatus. We provide novel predictions of secreted proteins in these two organisms, as well as in the human pathogen *C. trachomatis*. Better understanding of secreted virulence factors in pathogens will lead to new ways of combating important infectious diseases and provide understanding of the complex interaction between pathogen and host.

## Introduction

Gram-negative bacteria are a major cause of many human diseases and, due to the emergence of antibiotic resistance, development of new means to combat their infection is a goal of the world health organization (WHO) and other international health organizations [1]. Pathogenic bacteria express a large number of proteins associated with virulence some of which are secreted into the host milieu and interfere with normal host cell functions or immune response. Since many virulence factors allow the survival of pathogens under very specific infectious conditions they represent attractive targets for alternative therapies relative to current strategies, which aim to kill all bacteria and thus efficiently drive the emergence of antibiotic resistance and increase the host susceptibility to other infections by eliminating the normal flora [2].

The type III secretion system in Gram-negative bacteria forms the interface between the pathogen and its host [3],[4]. Electron microscopy has revealed that the secretion machinery forms a needle-like structure that spans the inner and outer bacterial membrane [4]–[6] and allows injection of protein effectors directly into the cytoplasm of the eukaryotic host cell [7]. Each bacterial species has a repertoire of effector proteins which enact the virulence program of the bacteria by directly interacting with host cell pathways [7]. Though some of the genes that comprise the secretion machinery are well-conserved between species [8],[9], sequences of virulence effectors are diverse and the identity and nature of their signal sequences, target protein(s) in the secretion complex, and methods of regulation are poorly understood [4].

While carboxy terminal sequences can be important, as a general rule secreted proteins are targeted to their cognate apparatus by a signal that is encoded in the N-terminal region of the protein or alternatively the 5′ end of the mRNA sequence, and provides a sequence-based signature for the system [10],[11]. To understand the type III secretion system and catalog its full complement of secreted substrates it is necessary to identify this secretion signal [4], [12]–[14]. Elucidation of the mechanism by which effectors are targeted to be secreted will provide valuable insight into the virulence program of many Gram-negative bacteria.

Effectors generally have two N-terminal domains that are important secretion. Residues 1–25 contain a region thought to be a secretion signal but that is highly variable in sequence [15] and, at least in some cases, highly tolerant of mutations [16],[17]. For some effectors this region has been shown to be both necessary and sufficient for secretion [10],[16],[18],[19]. However, no sequence motifs or common patterns have been identified that can be used to accurately predict type III secreted substrates. In addition, some effectors contain a chaperone binding domain that spans residues 25–100 [12]. Chaperones are necessary to stabilize some effectors, to maintain them in an unfolded state prior to secretion, and to expose the secretion signal sequence itself [4],[12]. It has been proposed that the N-terminal secretion signal is an ‘ancestral’ flagellar targeting signal and that the chaperone-binding domain and chaperone itself may in some cases target the effector to a specific secretion apparatus [19].

In this study we chose to analyze type III secreted effectors and their putative secretion signals in three organisms: *S.* Typhimurium, *P. syringae*, and *Chlamydia trachomatis*. Though all three organisms are Gram-negative pathogens with type III secretion systems, they differ in host range, evolutionary history [20], and lifestyles. Phylogenetic analyses of core components of the type III secretion systems also suggests that though they originated from a common ancestor, the secretion system from each of the organisms in this study falls into a different distinct group [21],[22]. Both S. Typhimurium and *P. syringae* have extensively characterized repertoires of type III secreted effectors [23],[24], which provide a sufficient number of examples for rigorous training and evaluation of a computational learning approach such as ours. *C. trachomatis* was chosen as an important pathogen, which has a relatively poorly defined, set of secreted effectors, and thus represents a good target for computational predictions [25].

Proteins secreted through the type III secretion system are highly variable in sequence. Though there are related families of effectors [26],[27], a significant number have no detectable sequence similarity to any other known effectors. Approaches based on sequence similarity, G+C content, genomic location within horizontally transferred regions of the chromosome, regulation by known virulence regulators, fusion to enzymatic or epitope tags, and homology between diverse pathogenic organisms have all been used to identify effectors with limited success [26], [28]–[35]. Most recently, a proteomic approach was used to greatly expand the estimated number of secreted effectors in pathogenic *E. coli* 0157:H7 [36]. This finding indicates that there are likely to be a large number of unknown effectors in type III secretion system-containing bacteria, even in well-studied organisms like *S.* Typhimurium and *P. syringae*. General features of the protein sequence have also been used to the same end, focused on the N-terminal secretion signal. In *P. syringae* the amino acid biases and patterns in the N-terminal secretion signal were used to identify novel effectors [30],[34],[37]. Detection of common promoter elements has also been used to identify novel effectors in *P. syringae* [32], but this approach is limited to known and detectable motifs. To date there have been neither systematic predictive studies of type III secretion system effectors nor a general strategy to identify proteins that are targeted to the type III secretion system.

We use a novel computational approach to identify secreted effectors based on sequence analysis and to delineate and define a putative N-terminal secretion signal common to the majority of type III secreted effectors. Our method, the SVM-based Identification and Evaluation of Virulence Effectors (SIEVE), is trained on a set of known examples of secreted effectors based on sequence-derived information and then used to provide accurate predictions of secreted effectors in evolutionarily distinct bacteria. We show that SIEVE can identify known secreted effectors very well with simultaneous specificity and sensitivity of greater than 88% for prediction of effectors when trained on one species and tested on the other, in the absence of detectable sequence similarity between effectors in the two sets. A considerable strength of our findings comes from the fact that we considered a large number of different sequences from effectors in multiple organisms. Previously this has only been used for detection of sequence homology between effectors using traditional approaches [36]. Our novel analyses allowed us to detect the presence of a protein-encoded secretion signal in the N-terminal 16–20 residues of the majority of type III secreted effectors examined. Though variable in sequence, we define the most important residues for this secretion signal across multiple organisms. Finally, we use a model trained on the effectors from *S.* Typhimurium and *P. syringae* to suggest new candidates for type III secretion in *S.* Typhimurium and in *C. trachomatis*, the most common cause of female infertility in the US [38].

## Methods

### Organisms Targeted and Datasets Used

We chose to target *S.* Typhimurium and *P. syringae* for our initial analysis because they have been well studied, especially in regard to type III secreted effectors, providing enough well-validated examples to train and evaluate our methods. *C. trachomatis* was chosen as a target for novel predictions because of the difficulties associated with studying it experimentally and its corresponding lack of well-validated secretion substrates.

*Salmonella* infection is a major public health problem with three million cases of infection per year in the U.S. alone [39]. With the recent emergence of untreatable, multi-drug resistant strains such as phage type DT104 [40] the public health threat has become greater. Genome sequences were obtained from the NCBI database for *S.* Typhimurium LT2 (AE006468) and associated virulence plasmid (AE006471). A set of 36 *S.* Typhimurium proteins reported to be type III secreted effectors was compiled from the literature (Table 1; see also [23]).

**Table 1:** Known secreted effectors used for training SIEVE and their scores using the STM to STM and PSY to STM SIEVE models.

ID	Name	Description	System	STMtoSTM	Rank	PSYtoSTM	Rank
STM1055	gtgE	Gifsy-2 encoded effector	???	4.06	*1*	2.20	*12*
STM0972	sopD-2	homologous to secreted protein sopD	SPI-2	3.82	*2*	1.80	*26*
STM1051	sseI/srfH	Secretion system effector	SPI-2	3.46	*3*	1.81	*25*
STM1583	steA	putative cytoplasmic protein	both	3.29	*4*	1.94	*20*
STM1088	pipB-1	Pathogenicity island encoded protein: SPI5	SPI-2	3.23	*5*	2.05	*16*
STM1631	sseJ	Salmonella translocated effector	SPI-2	3.11	*6*	1.79	*27*
STM2945	sopD-1	secreted protein in the Sop family	SPI-1	3.09	*7*	1.27	*33*
STM1602	sifB	Salmonella translocated effector	SPI-2	3.06	*8*	1.97	*19*
STM4157	sseK-1	putative cytoplasmic protein	SPI-2	3.05	*9*	1.53	*31*
STM2137	sseK-2	putative cytoplasmic protein	SPI-2	3.01	*10*	2.00	*17*
STM1224	sifA	replication in macrophages	SPI-2	2.97	*11*	1.90	*21*
STM2584	gogB	Gifsy-1 prophage: leucine-rich repeat	both	2.86	*12*	1.61	*29*
STM2614	gogA	Gifsy 1 encoded effector	???	2.68	*13*	1.29	*32*
STM2865	avrA	putative inner membrane protein	SPI-1	2.66	*14*	1.89	*23*
STM2884	sipC	cell invasion protein	SPI-1	2.62	*15*	2.20	*11*
**STM1404**	**sseF**	**Secretion system effector**	**SPI-2**	**2.56**	***16***	**2.61**	***5***
STM1855	sopE-2	TypeIII-secreted protein effector	SPI-1	2.49	*17*	1.87	*24*
STM2878	sptP	protein tyrosine phosphate	both	2.42	*18*	1.73	*28*
STM1398	sseB	Secretion system effector	SPI-2	2.38	*19*	2.06	*15*
**STM2066**	**sopA**	**Secreted effector protein**	**SPI-1**	**2.37**	***20***	**2.53**	***6***
STM1026	gtgA	Gifsy 2 encoded effector	???	2.34	*21*	1.60	*30*
**STM2883**	**sipD**	**cell invasion protein**	**SPI-1**	**2.33**	***22***	**2.70**	***3***
STM1393	ssaB/spiC	Secretion system apparatus	SPI-2	2.22	*23*	2.14	*13*
**STM2868**	**orgC**	**secreted repressor**	**???**	**2.11**	***24***	**2.33**	***9***
**STM2892**	**invJ**	**surface presentation of antigens**	**SPI-1**	**2.03**	***25***	**2.90**	***2***
**STM1091**	**sopB/sigD**	**homologous to ipgD of Shigella**	**SPI-1**	**1.99**	***26***	**2.66**	***4***
**STM1400**	**sseC**	**Secretion system effector**	**SPI-2**	**1.94**	***27***	**3.13**	***1***
STM1402	sseE	Secretion system effector	SPI-2	1.91	*28*	1.20	*34*
STM1401	sseD	Secretion system effector	SPI-2	1.88	*29*	1.98	*18*
**STM1405**	**sseG**	**Secretion system effector**	**SPI-2**	**1.78**	***30***	**2.51**	***7***
STM2780	pipB-2	homologue of pipB	SPI-2	1.52	*31*	0.45	*36*
STM2882	sipA	cell invasion protein	SPI-1	1.40	*32*	2.12	*14*
STM0800	slrP	leucine-rich repeat protein	both	1.37	*33*	1.90	*22*
STM2885	sipB	cell invasion protein	SPI-1	1.25	*34*	2.32	*10*
**PSLT039**	**spvB**	**Salmonella plasmid virulence**	**???**	**1.17**	***35***	**2.38**	***8***
STM2241	sspH-2	Leucine-rich repeat protein	SPI-2	1.11	*36*	1.16	*35*

The top 10 highest scores from the PSY to STM model are shown in bold.

*P. syringae* strains have a broad host range in plants and cause a variety of diseases and is an important model system in plant pathology. Numerous studies of the secreted effector repertoires in *P. syringae* have been published [30],[32],[34],[37],[41],[42]. This makes it an attractive model organism for testing methods to predict secreted effectors. We used the genome sequence from NCBI for *P. syringae* pathovar phaseolicola (NC_005773) and a set of 32 *P. sryingae* type III secreted effectors was downloaded from the Pseudomonas-Plant Interaction website (http://www.pseudomonas-syringae.org/) hypersensitive response and pathogenicity (Hrp) outer protein (hop) virulence protein database.

*C. trachomatis* is an obligate intracellular pathogen infecting humans and causes a variety of sexually transmitted diseases [43], as well as trachoma, a leading cause of preventable blindness worldwide [44]. The Chlamydiae infect a wide range of vertebrates and free-living amoebae and are a considered to be only distantly related to the Proteobacteria [22]. Though the genome sequence of *C. trachomatis* revealed the presence of a type III secretion system [45], research on this system and its effectors has lagged due to difficulty cultivating this genetically intractable, obligate intracellular pathogen [14]. We obtained the genome sequence of C. trachomatis (AE001273) from the NCBI database.

SIEVE predictions for all proteins in these organisms as well as *Shigella flexneri*, *Yersinia pestis* and *Vibrio cholerae*, is available as Table S5.

### Removal of Effector Homologs Identified by BLAST

To accurately determine the performance of SIEVE across organisms, all effectors in *P. syringae* that had any level of sequence similarity detectable by BLAST [46] to any effector in *S.* Typhimurium were removed. This reduced the number of effectors used in *P. syringae* from 32 to 29, eliminating HopAN1, HopAJ1 and HopAJ2 from consideration. BLAST was executed with default parameters meaning that sequence matches with expectation values worse than 2.0 were not reported. This process provides a conservative group of non-redundant effectors, ensuring that the performance results we report are not based on sequence similarity.

### Machine-Learning Methodology

Support vector machines (SVM) are a class of computational algorithms for classification [47],[48]. Essentially, they can learn patterns based on known members of a class of protein sequences (positive examples) and the corresponding protein sequences, which are not members of that class (negative examples). This process is referred to as “training” the algorithm and results in a computational “model”. The model can then be used to classify a different set of known examples to evaluate the performance of the model or can be applied to a set of unknown sequences to provide novel predictions. Information from each example sequence is used to train the model and the particular types of information chosen are referred to as the “features” of the model.

For training the SVM in SIEVE we chose to use known secreted effectors as positive examples and proteins that have not been identified as effectors, i.e. the remainder of the proteins in the organism, as negative examples. The true set of negative examples is actually unknown; in fact we show that a number of the proteins in our negative example set are secreted but had not been identified during compilation of our initial positive example set. This fact means that the performance we report using SIEVE is a conservative, lower bound estimate, since it contains an unknown number of misclassified false-positive predictions (i.e. real secreted effectors that have not yet been discovered).

Features are the different sequence characteristics used as input to the SVM. The SVM uses the features to learn the difference between the positive and negative examples. Five sets of features were chosen for SIEVE based on their known or suspected distributions in secreted effectors: evolutionary conservation of the protein sequence (CONS), a phylogenetic profile of sequence similarity to 54 other genomes (PHYL; Table S1), nucleotide composition of the cognate gene (GC)[35], amino acid composition (AA)[17],[41],[49],[50], and finally the sequence of the N-terminal 30 residues of the protein sequence (SEQ)[30],[50]. To determine the most important features for classification we used an iterative process known as recursive feature elimination (RFE) that successively eliminates features with low impact on the overall performance of the model.

We used the SVM software suite Gist [51] to perform all training, testing and evaluation of different models. Except where noted (e.g. Figure S1), we used a radial basis function kernel with a width of 0.5 and an optimized ratio of negative to positive examples (Figure S2) for SIEVE classification. See Text S1 for further details on machine-learning methods and the evaluation approaches used.

### Performance Evaluation

To evaluate the performance of the method we used measures of sensitivity, the number of predictions that were correctly predicted as true positives divided by the number of all positive examples (TP/(TP+FN)), and specificity, the number of predictions that were correctly predicted as true negatives divided by the number of all negative examples (TN/(FP+TN)). We also used a common measure of performance for classification tasks, the receiver operating characteristic (ROC) curve that is produced by plotting the sensitivity of the method versus specificity [52]. The area under a ROC curve (AUC) is 1 when all examples (positive and negative) are classified correctly and is 0.5 when classification is random.

## Results/Discussion

### Existing Methods for Computational Identification of Type III Secreted Effectors

Bioinformatics approaches have been used to identify secreted effectors in a variety of organisms with some success [30],[34],[36],[37]. However, the approaches described in these studies are focused on predicting effectors in a single organism and do not generalize to prediction in other organisms or are based on homology with known effectors. Accordingly, we wanted to test the ability of these methods in predicting secreted effectors in *S.* Typhimurium.

We first examined the ability of SecretomeP [53], a program which identifies non-classically secreted proteins generally in Gram-negative bacteria (http://www.cbs.dtu.dk/services/SecretomeP/). SecretomeP identified 12 of 36 known effectors in *S.* Typhimurium to be secreted, but also identified over 400 non-type III secreted proteins, yielding an overall precision of less than 3% for type III secreted substrates. This is not surprising since the method is trained on proteins secreted by a number of different systems, and is not designed to specifically identify type III secreted effectors.

We next tested the ability of two simple measures to discriminate secreted effectors; the G+C content of the associated gene and the number of number of polar residues [34] in the N-terminal 30 amino acids of the protein. Plotting the sensitivity of this method versus its specificity gives the receiver operator characteristic (ROC) curve, which provides a summary of the performance of a method to classify things into two categories. Surprisingly, we found that the G+C content gave performance of 0.89 (as judged by ROC analysis) to discriminate secreted effectors from other proteins in S. Typhimurium. However, even with this performance the top 5 true positive predictions could be discriminated with a precision of only about 6% (i.e. with 81 false positive predictions) so the level of precision possible using this measure alone was also low. Additionally, we found that G+C content gave an ROC of 0.73 for prediction of *P. syringae* effectors indicating that it cannot be used to identify all effectors with the same confidence. The observed performance of G+C content in *S.* Typhimurium may be due to the fact that most effectors are located in horizontally transferred pathogenicity islands or islets, such as SPI-1 and SPI-2 [54],[55]. Amino acid biases were largely uninformative for predicting effectors but the count of serine residues in the N-terminal 100 residues gave an ROC of 0.73. This is consistent with previous observations of amino acid biases, including serine, in the N-terminal regions of effectors [24],[41].

One previously published study that identified secreted effectors in *P. syringae* based in part on bioinformatics techniques [30] defined two sequence motifs. Secreted effectors were predicted by first searching for these two motifs then applying several other heuristic rules (e.g. sequences shorter than 150 residues were screened out). We applied these same set of criteria to *S.* Typhimurium proteins and found that they could correctly identify only two of the known secreted effectors out of a total of 52 predictions (4% precision). This shows that these patterns while accurate on *P. syringae* are not applicable to *S.* Typhimurium.

Another recent study used BLAST-determined sequence similarity between secreted effectors in different organisms to identify novel secreted effectors in *Escherichia coli* O157:H7 [36]. Though this approach is applicable to identification of secreted effectors in other organisms, it is based on detectable sequence similarity between known effectors, which is a significant limitation. The performance of the BLAST-based approach (see Text S1) was 0.79 for prediction of known effectors in *S.* Typhimurium. Nearly one-third of the known effectors in *S.* Typhimurium showed no detectable sequence similarity to any of the effectors in the compiled list of all known effectors and thus could not be identified by this approach.

Our results from applying these previously described methods for identification showed that though G+C content alone was surprisingly effective at predicting secreted effectors, its precision was too low to provide very useful predictions. Likewise, sequence patterns developed in *P. syringae* and more general amino acid composition biases provide limited discrimination. Finally, BLAST similarity to known secreted effectors in other organisms provided reasonable discrimination, but this approach identified only those secreted effectors that have been identified in another organism.

### Prediction of Type III Secreted Effectors Using SIEVE

We found that existing computational methods to identify secreted effectors were somewhat effective in different ways when applied to known effectors in *S.* typhimurium. We therefore wanted to see if the integration of some of the data underlying these approaches could be used for more accurate prediction of secreted effectors. With this in mind we developed an approach to integrate genomic sequence information using computational techniques from data integration and machine learning techniques (the SVM-based Identification and Evaluation of Virulence Effectors or SIEVE). Similar methods have been used successfully for various classification tasks using biological sequences [56]–[66]. These methods use a set of known training examples to classify novel examples based on a set of features derived from the gene and/or protein sequences. We chose to integrate several features, using numeric values derived from analysis of the protein sequence, that have been directly or indirectly suggested to be important in discrimination of secreted effectors by previous studies from a number of organisms [17],[30],[35],[41],[49],[50]. These include the G+C content (GC) and general amino acid biases (AA), shown to have predictive value individually (see above) as well as evolutionary relationships (EVOL and PHYL). Finally, we included the N-terminal sequence of proteins (SEQ) to allow the method to learn sequence patterns or biases that might be predictive of secreted effectors. The features used by the method are described in detail in Text S1.

**Figure 1:**
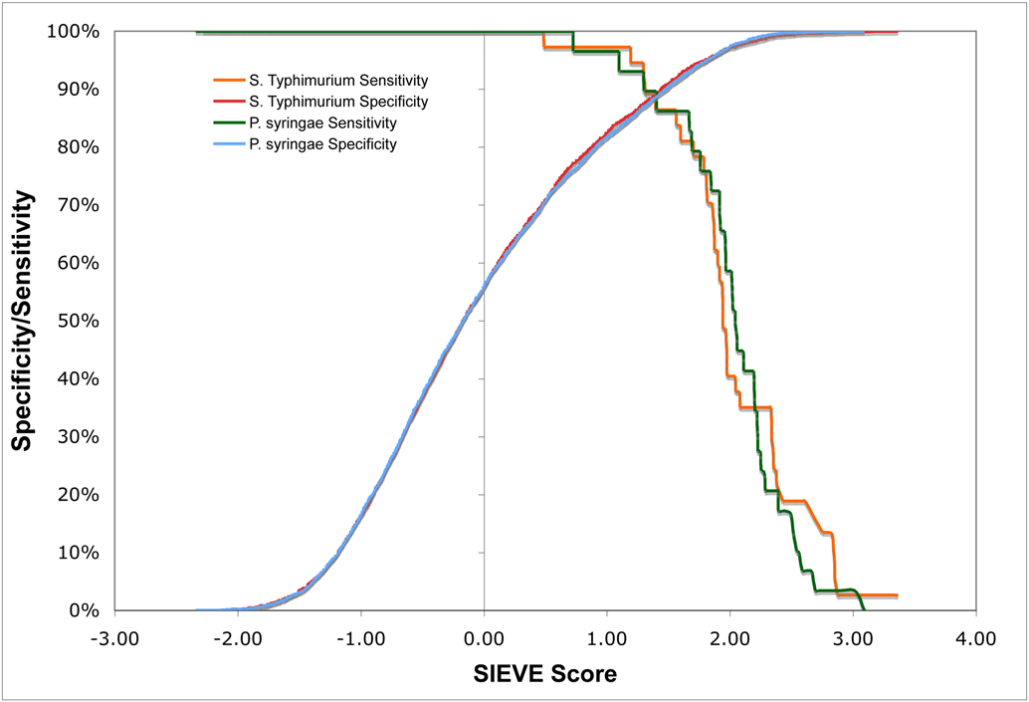
Accurate identification of type III secreted effectors using sequence data. The sensitivity (TP/(TP+FN); solid lines) and specificity (TN/(FP+TN); dashed lines) of SIEVE on *S.* Typhimurium predictions (PSY to STM model; red) and *P. syringae* (STM to PSY model; blue) effectors were calculated as a function of a SIEVE score threshold (X axis). The results show that both models perform well providing a maximum sensitivity and specificity at about 90%. For example 33 of 36 known *S.* Typhimurium effectors are in the top 10% of predictions.

To assess the ability of SIEVE to identify novel secreted effectors we trained a SIEVE model on the set of effectors from one organism then evaluated the methods performance on a set of effectors from a different organism that were not used in the training process. We examined the performance of a SIEVE model trained on *P. syringae* proteins and evaluated on *S.* Typhimurium proteins (PSY to STM) and the reverse experiment of SIEVE trained on *S.* Typhimurium proteins and evaluated on *P. syringae* proteins (STM to PSY). These results show that the SIEVE approach performs very well at classification in terms of both specificity and sensitivity (Figure 1). At a sensitivity of 90%, i.e. 33 *S.* Typhimurium effectors and 26 *P. syringae* effectors, the specificity of the method is 88% when used to predict *S.* Typhimurium effectors (PSY to STM model) and 87% when applied to *P. syringae* effectors (STM to PSY model). The performance (ROC) values for classification were 0.95 and 0.96, respectively. These results indicate that our approach to integration of the chosen sequence-based features using a non-linear classification method accurately predicts type III secreted effectors between distantly related organisms. This suggests that there may be a set of features that are shared between effectors in both organisms, a hypothesis that we tested next.

### Delineation of a Common Putative Secretion Signal

**Figure 2:**
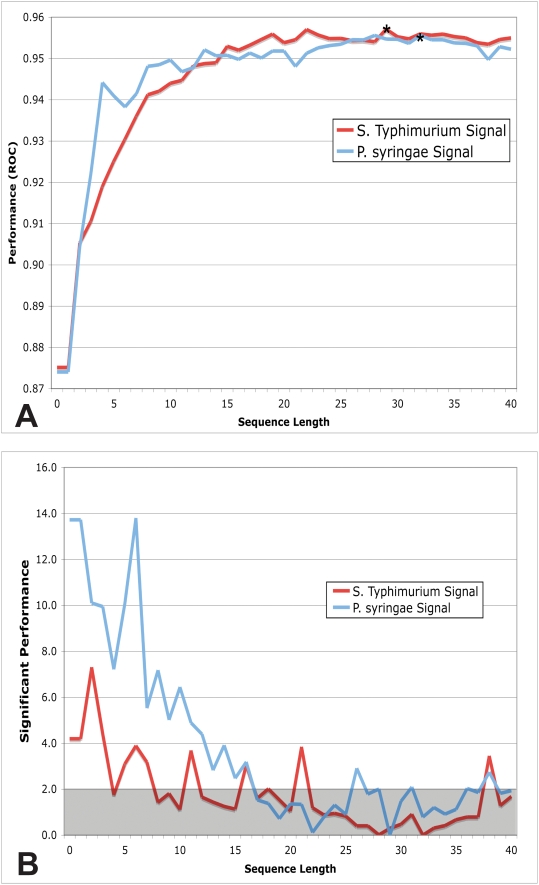
Delineating the length of the type III secretion signal. A. The performance of SIEVE on *S.* Typhimurium (PSY to STM model; red) and *P. syringae* (STM to PSY model; blue) was evaluated using the ROC area under the curve metric described in the text (Y axes). Models were trained using the indicated number of residues from the N-termini of the examples (X axis) and tested on the complete testing set (i.e. the entire set of positive and negative examples from the other organism). Maximum performance of both models was at approximately 30 residues (asterisks) suggesting that this might be the maximum length of a secretion signal. B. From the analysis in panel A we calculated the difference from the maximum ROC value (at 29 for the PSY to STM model and 32 for the STM to PSY model) for each length sequence and divided this by the standard error (difference from maximum, Y axis) for that sequence length (X axis). This shows the significance of each sequence length, with values below 2.0 (grey area) having insignificant differences (as judged using standard error). For *S.* Typhimurium effectors (PSY to STM model) the longest sequence length that is significantly different from the maximum value is 21 residues and for the *P. syringae* effectors (STM to PSY model) it is 16 residues. These lengths agree generally with previous estimates of secretion signal length.

Several studies have highlighted the importance of a short region in the N-termini of effectors in secretion [18],[67],[68]. This region, thought to be between 10 and 50 amino acids in length, has sometimes been referred to as the secretion signal, though it does not contain any recognizable sequence pattern. Because our models included N-terminal sequence information we wanted to determine the length of sequence that provided the maximum discriminatory power for classification. We therefore examined the effect of including sequences of different lengths in both models to provide accurate discrimination of effectors. We trained models with the other types of features (EVOL, GC, AA and PHYL) using the N-terminal 0 to 40 residues as the SEQ feature set. A total of 10 models for each sequence length were trained using randomly selected negative examples and the mean performance (i.e. ROC) was calculated. The results for the *S.* Typhimurium signal (PSY to STM model) and the *P. syringae* signal (STM to PSY model) are shown in Figure 2A. Both models show an increase in performance from the baseline value (which includes no SEQ features) reaching a maximum when the length of the sequence reaches 29 or 31 amino acid residues, respectively. Additional sequence information beyond this length does not improve the ability of the model to classify effectors in the opposite organism.

We next determined the sequence length that provides the majority of the information for each model, i.e. what is the length of sequence beyond which adding more residues to the model fails to improve performance significantly? This analysis is shown in Figure 2B and was performed by calculating the difference in performance between the maximum performance for that model and performance for each sequence length and dividing this number by the standard error for that performance. In this analysis values that are less than 2.0 represent insignificant differences, for which the standard error would begin to overlap from the two values. According to the plot in Figure 2B the maximum significant length for the N-terminal sequence was determined to be 21 and 16 for *S.* Typhimurium (PSY to STM model) and *P. syringae* (STM to PSY model) effectors, respectively.

These lengths agree generally with previously determined estimates of the length of the secretion signal [4],[12],[16],[18],[24],[67],[68] and indicate that a significant amount of information is shared between effectors across organisms in their N-terminal 30 residues, with most of the information residing in the first 16–20 residues. These results further support the hypothesis that there is a significant, sequence-based secretion signal in the N-termini of effectors which is not possible to detect using traditional alignment methods such as BLAST.

### Computational Identification of a Putative Secretion Signal

Based on the success of our models at accurately identifying secreted effectors from sequence information we examined the hypothesis that this region contains a hidden sequence motif, possibly derived from an ancient ancestor [19]. To determine the most important sequence-derived features for the classification task in each of the models we used a recursive feature elimination approach (see Text S1 for details). We found that a minimal set of 88 (out of a total of 711) features retained the ability to accurately classify secreted effectors (Figure S4). The features that are most important for accurate classification include the evolutionary conservation feature (CONS) and G+C content (GC), as well as several phylogenetic profile (PHYL) features (see Text S1) and a number of specific sequence biases that span the 30 residue putative secretion signal discussed below.

**Figure 3:**
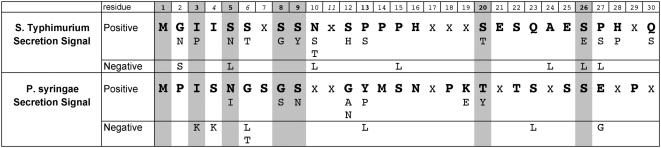
Identification of a shared sequence motif in type III secreted effectors. We identified the features (sequence locations and residue types) with the greatest ability to classify *S.* Typhimurium and *P. syringae* secreted effectors (see text and Figure S4). The residue type with the highest positive weight is shown in bold for each position, followed by the other residue types that were also found to be significant. Amino acids with a negative weight are also shown. Positions with an “x” have no representation in the minimal set. Grey background indicates sequence positions where both models agree (for at least one amino acid type). It is important to note that this does not represent a consensus sequence, since there is very little similarity between individual effector signals (see Table S4). Rather it shows those sequence positions and amino acid types that SIEVE found particularly helpful in discriminating between the secreted effectors and negative examples.

The models both contained a set of significantly important residues. These residues, shown in Figure 3, represent those positions and residue types that the models found to be most important for classification. They form two weak sequence motifs, which are detectable by SIEVE in comparison to the background the N-terminal sequences from all other non-secreted proteins in the organism. The most significant sequence features that are shared between the two models are also shown in Figure 3 with a grey background. This indicates that the secretion signal from both organisms are more likely to have an isoleucine at position 3, an asparagine at position 5, a serine or glycine at position 8, and a serine at position 9, in addition to several other shared biases. The concentration of shared important features in the N-terminal 10 residues agrees with results from the sequence length analysis (Figure 2) showing that the greatest gains in classification performance are from this region.

The sequence motifs obtained here are consistent with a number of previous observations. They are rich in polar residues, especially serines, and have few charged residues, as observed in *P. syringae* [24],[41]. The sequence patterns previously derived from *P. syringae* effectors [30] are almost completely consistent with the sequence biases from our models. Though, as we showed, these patterns are ineffective at accurately discriminating effectors in *S.* Typhimurium. Finally, it was shown that all proteins bearing synthetic secretion signals with the pattern MxIISSxS, among others, were highly secreted in *Yersinia pestis* [17], which agrees well with the pattern identified for *S.* Typhimurium.

Our results support the existence of a conserved, though highly variable, secretion signal encoded in the N-terminal 16–20 residues of type III secreted effectors. The important residues do not form a classic sequence motif but rather can be thought of as significant residue tendencies of the secretion signal. This type of secretion signal has been found in other secretion systems, most notably the Sec system in bacteria [69]. In the Sec system no specific sequence motif for secretion exists but a pattern of charged residues and a hydrophobic domain allows accurate detection of secreted substrates [70]. Collectively these results represent a large number of hypotheses that can be tested, for instance using mutagenesis and secretion assays, that will further elucidate the nature of the secretion signal and can help refine the models presented here. The lack of a classical sequence motif for secretion is expected from the historical failure of traditional sequence motif identification methods to identify type III secretion signals. It may also partly explain the observation that the N-terminal sequence shows considerable plasticity and yet can be functional [4],[16]. We provide the unaligned N-terminal sequences of the effectors used in this study and show their agreement with the sequence tendencies presented in Figure 3 as Table S4.

### Identification of Novel Putative Type III Secreted Effectors in *S.* Typhimurium

We next wanted to test if SIEVE could generate useful predictions of novel type III secreted effectors in a well-characterized bacteria. Accordingly, we generated a ranked list of predictions by combining results from two applicable models (PSY to STM and STM to STM, see Text S1) in *S.* Typhimurium. We show a selection of the highest scoring ∼2% of the predictions in Table 2, and the remainder of these predictions are available as Table S2. To help biologists interpret the scores associated with each prediction we calculated a confidence range for novel predictions based on a conservative set of positive and negative examples (those described here) and a “generous” set. The generous set uses a set of negative examples that limited to those proteins with well-defined functions. This process is described in Text S1 (Figure S3) and is used to provide useful hypotheses for experimental validation.

**Table 2:** High confidence secreted effector predictions in *S.* Typhimurium.

ID	Name	Description	Score	Confidence^1^	Reference^2^
**PSLT037** 3	**spvD**	**Salmonella plasmid virulence: hydrophilic protein**	**3.48**	**100%**	[**72**]
**PSLT038** 3	**spvC**	**Salmonella plasmid virulence: hydrophilic protein**	**2.35**	**70%**	[**71**],[80****]
STM1417	ssaP	Secretion system apparatus	2.97	100%	[81],[83]
STM2897	invE	invasion protein	2.60	85%	[80]–[82]
PSLT073	traM	conjugative transfer: mating signal	2.38	70%	
PSLT075	traJ	conjugative transfer: regulation	2.43	75%	
PSLT102	traS	conjugative transfer: surface exclusion	2.21	60%	
STM2087	rfbV	LPS side chain defect: abequosyltransferase	2.60	85%	[80]
STM2088	rfbX	LPS side chain defect: putative O-antigen transferase	2.38	70%	[80],[81]
STM1332	rfc	O-antigen polymerase	2.70	90%	
STM2112	wcaD	putative colanic acid polymerase	2.21	60%	
**STM1244** 3 ^,^ 4	**pagD**	**PhoP regulated**	**2.08**	50%	[81]
STM1867	pagK	PhoPQ-activated gene	2.47	75%	[81]
STM1240	envF	putative envelope lipoprotein	2.41	75%	[81]
STM2866	sprB	transcriptional regulator	2.16	55%	
STM1087	pipA	Pathogenicity island encoded protein: SPI3	2.30	70%	
STM1381	orf245	putative cytoplasmic protein	2.32	70%	[80],[81]
STM1896		putative cytoplasmic protein	2.19	55%	[80],[81]
STM2761		putative inner membrane protein	3.00	100%	[82]
**STM1668** 3 ^,^ 5	**zirS**	**putative outer membrane or exported**	**2.57**	**85%**	[**79]**,[82****]
STM4302		putative cytoplasmic protein	2.41	75%	[82]
STM4316		putative cytoplasmic protein	2.34	70%	[82]
STM1228		putative periplasmic protein	2.33	70%	[82]
STM3026		putative outer membrane protein	2.14	55%	[82]
STM2138		putative cytoplasmic protein	2.71	90%	[81]
STM2585A		Gifsy-1 prophage: similar to transpose	2.58	85%	[81]
STM4257		putative inner membrane or exported	2.49	80%	[81]
STM0284		putative shiga-like toxin A subunit	2.44	75%	[81]
STM2225		putative inner membrane protein	2.26	65%	[81]
STM1868A		putative protein	2.19	55%	[81]
STM1554		putative coiled-coil protein	2.59	85%	[80]
STM0100		putative cytoplasmic protein	2.54	80%	[80]
STM4155		putative inner membrane protein	2.39	70%	[80]
STM2208		putative inner membrane protein	2.35	75%	[80]
STM3052		putative outer membrane protein	2.28	65%	[80]

1 confidence based on the “generous” estimate in Figure S3.

2 references for secretion or involvement in virulence.

3 proteins experimentally determined to be secreted.

4 L. Crosa and F.H., unpublished results.

5 not secreted by a type III secretion system.

Investigating the proteins in Table 2, we found evidence that the SIEVE predictions identify proteins that are likely to be secreted. The SIEVE results for *S.* Typhimurium contain two highly confident predictions (SpvD and SpvC), which are in an operon that is co-regulated with SPI-2 and contains SpvB, which is a known effector. Though SpvC was not included in our positive example set a recent publication has identified it as being a secreted effector [71]. Although there was evidence that SpvD was secreted into the supernatant [72], these results did not show that it was a type III secreted effector and so SpvD was also not included in our positive example set. SpvD is the prediction with the highest score providing further evidence that it is a secreted effector. The prediction list also includes three proteins for which the cognate gene is regulated by the PhoP/Q two-component regulatory system [73]–[75], envF and pagDK. PhoP/Q is induced in acidic and Mg_2+_-poor medium and within the macrophage phagosome [76]–[78]. We used a CyaA fusion assay to show that PagD is secreted in macrophages (L. Crosa and F.H. unpublished results), further validating that the approach is useful for predicting secreted effectors. Finally, the ZirS protein was identified by SIEVE. Interestingly, this protein was recently found to be the secreted protein from a novel two-partner secretion system, ZirTS [79]. Though ZirS is thought to have a cleaved signal peptide directing it through the inner membrane our findings suggest that the targeting signal for ZirS may be similar to that of the type III secretion system. In total, four of our novel predictions have been shown to be secreted experimentally. We are currently validating other predictions.

Since many of our novel predictions do not have functional annotations and have not been experimentally investigated individually, we assessed the general role of proteins predicted to be secreted by SIEVE in virulence by one or more negative selection studies designed to detect genes essential for virulence *in vivo* [80]–[83]. From this analysis we found a greater than 2-fold enrichment of predictions implicated in one or more negative selection study in the predictions with scores in the top 10% relative to those in the remaining 90% (p value 1e-28; using a two-tailed Student's t-test). It is important to note that many of the known *S.* Typhimurium effectors (10 of 37) were not identified in any of the original negative selection experiments most likely due to functional redundancy as well as specifics of the virulence assay employed in terms of different hosts and/or cell types. So the fact that some of our predictions are not found on these lists does not mean that they are not important in virulence. Rather, predictions that are known to be essential in virulence represent high-priority targets for future investigation.

Two classes of genes identified appear to be false positive predictions. Several components involved in the biosynthesis of lipopolysaccharide (LPS) and O-antigen are identified by SIEVE. Since the complex directing biosynthesis and transport of LPS occurs at the inner membrane [84], it is possible that components of this system use a targeting signal that is similar to type III secreted effectors. Several plasmid-encoded conjugative transfer proteins are also identified by SIEVE; TraJ, TraM, and TraS. The conjugative transfer system transfers a nucleoprotein complex during mating pair formation [85]. The TraM and TraJ proteins are associated with the relaxosome [86], the protein complex that binds DNA and readies it for transport through the associated type IV secretion system [85] and TraS is an outer membrane protein involved in the entry exclusion (Eex) system. It is possible that components of the type IV secretion system may share some similarity with the type III system that allows them to be identified by SIEVE.

SIEVE predicted components from three different functional groups to contain secretion signals, type III secretion system substrates, type IV secretion system-associated complexes and LPS biosynthesis proteins. Each of these are targeted to the cytoplasmic face of the inner membrane, either to be secreted or to form a functional complex. Our findings imply that diverse mechanisms of membrane targeting may share common features that direct targeting. Though they have different mechanisms, the types III and IV secretion systems share the common function of transporting virulence factors into host cells. The similarity between these two systems is supported by the observation that some type IV secreted effectors in *Legionella pneumophila* can be identified using SIEVE trained on type III secreted effectors from *S.* Typhimurium (J.M. unpublished results).

As can be seen in Table 2, a number of other interesting predictions are made by SIEVE. However, the value of the SIEVE approach is demonstrated in that 74 of the predictions (82%) have unknown or poorly described functions. Of these proteins 19 have been implicated in virulence by at least one of the negative selection studies, providing a reasonable starting point for experimental investigation.

### Identification of Novel Putative Type III Secreted Effectors in *C. trachomatis*

Finally, we examined the ability of SIEVE to provide useful predictions of type III secreted effectors for an organism that is difficult to study. We trained SIEVE on the positive and negative examples from both *S.* Typhimurium and *P. syringae* and applied the model to the *C. trachomatis* genome. Examining the list of top 10% of predictions (Table 3) from *C. trachomatis* showed that a number of these proteins have been demonstrated to be secreted (bold type) by various experimental methods or predicted to be secreted by other computational approaches.

**Table 3:** Predicted secreted effectors in *C. trachomatis*.

ID	Name	Description	Score	Confidence^1^	Reference
CT006	-	hypothetical protein	1.32	20%	[25],[90]
CT007	-	hypothetical protein	1.41	25%	
CT011	-	hypothetical protein	1.63	40%	
**CT049**	**pls1**	**hypothetical protein**	**1.89**	**40%**	[**92**]
**CT050**	**pls2**	**hypothetical protein**	**2.26**	**65%**	[**90]**,[92****]
CT060	flhA	Flagellar Secretion Protein	1.59	30%	
CT080	ltuB	hypothetical protein	1.90	50%	
CT082	-	hypothetical protein	2.57	80%	
CT087	malQ	4-alpha glucanotransferase	2.10	50%	
CT088	sycE	Secretion Chaperone	1.93	50%	
**CT089**	**lcrE/copN**	**Low Calcium Response E**	**1.75**	**40%**	[**90]**,[91],[105****]
CT101	-	hypothetical protein	1.40	25%	[25]
CT105	-	hypothetical protein	3.43	100%	
**CT118**	**-**	**hypothetical protein**	**1.38**	**20%**	[**90**]
CT142	-	hypothetical protein	1.36	20%	
**CT147**	**-**	**hypothetical protein**	**1.80**	**40%**	[**90**]
CT148	mhpA	Monooxygenase	1.41	25%	
**CT154**	**-**	**Phospholipase D Endonuclease**	**2.10**	**50%**	[**93**]
**CT155**	**-**	**Phospholipase D Endonuclease**	**1.53**	**30%**	[**93**]
**CT157**	**-**	**Phospholipase D Endonuclease**	**2.42**	**75%**	[**93**]
CT164	-	hypothetical protein	2.18	60%	[25],[90]
CT165	-	hypothetical protein	1.67	40%	
CT166	-	hypothetical protein	1.91	50%	
CT174	-	hypothetical protein	1.30	20%	
CT181	-	hypothetical protein	2.40	70%	
CT196	-	hypothetical protein	1.55	30%	[25],[90]
CT198	oppA_3	Oligopeptide Binding Protein	1.33	20%	
CT205	pfkA_1	Fructose-6-P Phosphotransferase	1.44	25%	
CT214	-	hypothetical protein	1.44	25%	[90]
**CT223**	**-**	**hypothetical protein**	**1.40**	**25%**	[**25]**,[90****]
**CT228**	**-**	**hypothetical protein**	**2.61**	**85%**	[**25]**,[90****]
**CT229**	**-**	**hypothetical protein**	**1.96**	**50%**	[**25]**,[90****]
**CT232**	**incB**	**Inclusion Membrane Protein B**	**1.45**	**25%**	[**25]**,[90],[105****]
**CT233**	**incC**	**Inclusion Membrane Protein C**	**2.38**	**70%**	[**25]**,[90],[105****]
CT262	-	hypothetical protein	1.37	20%	
CT273	-	hypothetical protein	1.33	20%	
CT288	-	hypothetical protein	1.38	20%	
CT309	-	hypothetical protein	1.35	20%	
CT311	-	hypothetical protein	1.63	40%	
CT326	-	hypothetical protein	2.46	75%	
CT344	lon	Lon ATP-dependent protease	1.72	40%	
CT345	-	hypothetical protein	1.81	40%	[25],[90]
**CT358**	**-**	**hypothetical protein**	**2.03**	**50%**	[**25]**,[90****]
CT365	-	hypothetical protein	2.44	75%	[90]
CT384	-	hypothetical protein	1.78	40%	
CT391	-	hypothetical protein	1.31	20%	
CT392	yprS	hypothetical protein	2.36	70%	
CT412	pmpA	Putative outer memb. protein A	1.97	50%	
**CT440**	**-**	**hypothetical protein**	**1.68**	**40%**	[**25]**,[90****]
CT449	-	hypothetical protein	1.78	40%	[25],[90]
**CT456**	**TARP**	**hypothetical protein**	**2.81**	**90%**	[**94**]
CT461	yaeI	Phosphohydrolase	1.69	40%	
CT483	-	hypothetical protein	2.12	55%	[25],[90]
**CT529**	**-**	**hypothetical protein**	**1.53**	**30%**	[**89]**,[90****]
CT552	-	hypothetical protein	1.40	25%	
CT559	yscJ	Yop proteins translocation	1.35	20%	
**CT578**	**-**	**hypothetical protein**	**3.31**	**100%**	[**89**]
CT583	gp6D	CHLTR Plasmid Paralog	1.81	40%	
CT616	-	hypothetical protein	2.19	55%	
CT620	-	hypothetical protein	1.97	50%	
CT622	-	CHLPN 76 kDa Homolog	1.92	50%	
CT623	-	CHLPN 76 kDa Homolog	1.69	40%	
**CT642**	**-**	**hypothetical protein**	**1.43**	**25%**	[**89**]
CT664	-	adenylate cyclase-like protein	1.35	20%	
CT668	-	hypothetical protein	1.37	20%	
**CT671**	**-**	**hypothetical protein**	**1.76**	**40%**	[**89**]
CT672	fliN	Flagellar Motor Switch	1.74	40%	
CT694	-	hypothetical protein	3.52	100%	
CT695	-	hypothetical protein	2.28	65%	
CT696	-	hypothetical protein	1.38	20%	
CT711	-	hypothetical protein	1.98	50%	
**CT718**	**-**	**hypothetical protein**	**2.16**	**55%**	[**89**]
CT728	-	hypothetical protein	1.36	20%	[25],[90]
CT736	ybcL	hypothetical protein	2.75	90%	
CT794.1	-	hypothetical protein	1.50	30%	
CT795	-	hypothetical protein	1.96	50%	
CT809	-	hypothetical protein	1.79	40%	
**CT847**	**-**	**hypothetical protein**	**2.21**	**60%**	[**95**]
CT849	-	hypothetical protein	1.93	50%	
CT853	-	hypothetical protein	1.32	20%	
**CT860**	**-**	**hypothetical protein**	**1.92**	**50%**	[**89**]
**CT863**	**-**	**hypothetical protein**	**1.59**	**40%**	[**89**]
CT867	-	Membrane Thiol Protease	3.02	100%	
CT868	-	Membrane Thiol Protease	4.17	100%	
CT870	pmpF	Putative Outer Membrane Protein	1.65	40%	
CT872	pmpH	Putative Outer Membrane Protein	1.33	20%	

1 confidence based on the “generous” estimate in Figure S3.

Bold type indicates that the protein has been shown to be secreted in one of several experimental systems.

Because it is complicated to work with both in terms of culturing and genetic manipulation [14],[22], a number of studies have been performed to identify candidate effectors by expression in heterologous systems or in cell culture systems [87]–[90]. Several of these studies have identified candidate effectors by their localization in the host cell [90]–[92]. During infection Chlamydia resides in a specialized cytoplasmic vacuole, also called an inclusion. Thus proteins that are localized to the inclusion body membrane, as well as those that are present in the cytoplasm are thought to be secreted through the type III secretion system. A recent study investigated 50 Chlamydial proteins believed to be localized to the inclusion membrane based on previous experimental or predictive studies [90]. Twenty-two of these proteins were determined to be inclusion localized, and 12 of these appear on our high-confidence list. Also, none of the 7 proteins found to be not secreted by this study were predicted by SIEVE. A family of several phospholipase D-like proteins predicted by SIEVE have also been implicated in pathogenesis, though have not been shown to be secreted and/or localized to the inclusion body [93]. Finally, two polymorphic membrane protein (Pmp)-like proteins, Pls1 and Pls2, were found to be localized to the inclusion membrane [92]. However, their secretion was not blocked by a type III secretion system inhibitor, suggesting that they are secreted by a novel mechanism. Our findings suggest that, similar to the ZirS protein identified in *S.* Typhimurium, the secretion signals for Pls1 and Pls2 are related to the type III secretion signal.

A number of other proteins on our list were shown to be secreted by heterologous expression systems. One large scale study in *Shigella flexneri* [89] used a reporter system to identify 18 candidate secreted substrates, 7 of which are on our high confidence list. Other experiments identified TARP (CT456) [94] and CT847 [95] as secreted proteins, also showing that they were localized to the host cell during infection. Finally, our confident predictions include 8 proteins predicted to be secreted by a previous computational analysis [25], but not yet experimentally validated. Again, a large number of the predictions are hypothetical proteins with no annotation providing a specific and confident set of candidates for further study.

We also examined the known or predicted effectors that were not in the top 10% of predictions (Table S3). These included 21 proteins known to be secreted, but eight of these (including IncA) were in the top 30% of SIEVE predictions. It is important to note that some of the experimental methods used to identify secreted proteins, such as secretion in a heterologous system [89], are merely suggestive that the protein is secreted *C. trachomatis*. Therefore this list is likely to be both incomplete and contain a number of false positives.

In total, 24 of the 86 top SIEVE predictions (28%) are known secreted effectors, have been shown to be localized to the inclusion membrane or cytoplasm of the host, or have been shown to be secreted in a heterologous expression system. This is in contrast to the 21 of 788 (3%) of these proteins in the remaining 90% of the genome. We determined the performance of the method in *C. trachomatis* as 0.89, though this is a conservative estimate of since it is likely that this list is incomplete and may contain false positives. These results show that our method, trained on proteins from other organisms, can provide useful predictions for other bacteria.

### Conclusions

Identification of the secretion signal that allows proteins to be targeted for secretion is of paramount importance for understanding any secretion system [69]. The type III secretion system is essential for virulence in a number of pathogenic bacteria and has been well studied in terms of its regulation, structural organization and secreted substrates [4],[8],[9],[12]. Despite extensive investigation the nature and even existence of a secretion signal for substrates of the type III secretion system remains a debated topic [4]. Though the N-terminal region of a number of substrates has been shown to be necessary and, in some cases, sufficient, for secretion [16],[18], there is no clear sequence motif that is common to substrates, even those from the same bacteria. Several alternative hypotheses have been presented to explain this observation: that a cryptic amino acid sequence serves as the signal by adopting an unstructured or flexibly structured conformation; that the secretion signal is encoded by the mRNA and is not directly dependent on the protein sequence; or that targeting is accomplished by chaperone proteins that specifically bind the substrates [4]. There is evidence for each of these hypotheses indicating that targeting may be a complex and multifaceted process. Using an *in silico* approach, we provide evidence that the protein sequence in the N-terminal 30 residues of the majority of known substrates from two bacteria provides enough information to allow accurate classification by a machine-learning algorithm. We also show that there are significant sequence biases in this region, some of which are shared between organisms, but these are not identifiable by traditional sequence analysis methods. These findings indicate that there is a secretion signal encoded by the N-terminal 30 residues of type III secreted substrates and provide a number of testable hypotheses regarding this putative signal. Our results do not disprove the alternatives (that the signal is encoded by mRNA or resides on the chaperones) but indicate that a majority of the secreted substrates from *S.* Typhimurium and *P. syringae* have a protein-encoded, N-terminal secretion signal.

Most of the core components of the type III secretion system are conserved between species [22],[96] and several lines of evidence indicate that the targeting mechanisms employed by the system may also be conserved. The first is that type III secretion systems can export proteins encoding secretion signals from other bacteria [97]–[99]. The second is that a recently discovered class of type III secretion inhibitor can block secretion in *Y. pestis*, *C. trachomatis* and *S.* Typhimurium [2], [100]–[103], though the mechanism of inhibition is unclear [103]. Finally, it has been shown from available structures of effectors bound to their cognate chaperones that the structure of this interaction is conserved across species [104]. These observations have important implications for development of new antibacterial agents. Our findings support a model of the type III secretion process that includes a targeting mechanism that is conserved between organisms. Despite our identification of some generally conserved features between the two targeting signals the lack of a well-defined targeting sequence leaves open the questions of what features of the secretion signal are recognized by the targeting system. The importance of particular residues as determined by our analysis suggests that there are underlying conserved structural requirements which form the basis for recognition by the secretion apparatus but can be encoded by a large range of different sequences.

The results we have presented show only that there is a significant amount of shared information between secreted effectors, especially in the N-terminal sequences. We propose that this shared information represents a biological function, that of type III secretion, and that the sequence patterns identified are functionally important in terms of targeting the proteins to the secretion apparatus. Further experimental investigation, for example mutational analyses based on these predictions, is necessary to validate this hypothesis. Also, we have presented a large number of high-confidence predictions of novel secreted effectors, a number of which have experimental evidence strongly suggesting that they are secreted substrates. Again, further experimental investigation of these predictions will allow refinement of the approaches described.

The results presented here support previous findings that diverse bacteria have similar type III secretion system targeting signals [97]–[99]. We have described a novel computational method for data integration and classification to discriminate secreted effectors from the type III secretion system in two evolutionarily distinct organisms. The excellent performance of this method on discrimination of secreted effectors shows that the groups of effectors from the two organisms are similar based on a number of sequence-derived characteristics, despite the lack of detectable sequence similarity between them. Using the models produced by SIEVE we identified a set of conserved sequence biases that define a putative, common secretion signal for type III secreted effectors. This approach is a novel and effective way to identify secreted effectors in a broad range of pathogenic bacteria and provides valuable insight into the nature of the type III secretion signal.

## Supporting Information

Figure S1 Performance of SIEVE models using different SVM kernel functions and parameters. The performance of the PSY to STM (red) and STM to PSY (blue) models were evaluated using the ROC area under the curve metric described in the text (Y axis). Performance using the radial basis function (radial) with a width parameter of 0.5 provided the best results for both models.
(0.20 MB TIF)

Figure S2 SIEVE performance using different ratios of negative top positive examples in the training process. The performance of the PSY to STM (red) and STM to PSY (blue) models were evaluated using the ROC area under the curve metric described in the text (Y axis). Models were trained with the radial basis function kernel and a width of 0.5 (see Figure S1) using the indicated ratio of negative to positive examples (X axis) and tested on the complete testing set (i.e. the entire set of positive and negative examples from the other organism). ‘Natural’ indicates that the entire set of negatives (all the proteins in the organism) were used for training. Error bars indicate +/−1 standard error calculated from 10 training runs using random selections of negative examples. The best performance is obtained using ratios of 20∶1 and 60∶1 for the STM to PSY and PSY to STM models, respectively. For consistency the ratio of 20∶1 was chosen for further SIEVE training in both models since it gives the best performance for the PSY to STM model and reasonable performance for the STM to PSY model.
(0.17 MB TIF)

Figure S3 Estimation of SIEVE Prediction Confidence. A. The positive predictive value, the number of true positive predictions divided by the total number of predictions made at a particular score threshold (TP/(TP+FP)) is shown (Y axis) plotted against the SIEVE score threshold (X axis) for the conservative evaluation set, all proteins not experimentally determined to be secreted effectors are treated as negatives (green line), and the generous evaluation set, only proteins with known functions are considered as negative examples (black line). B. The number of false positive predictions (green; with known functions), novel predictions (grey; with no known function) and known secreted effectors (blue) are shown at several different confidence thresholds (as determined from the generous evaluation set).
(3.00 MB TIF)

Figure S4 A minimal set of sequence-based features for accurate discrimination of type III secreted effectors. A recursive feature elimination approach was used that successively eliminates the 50% of the features in the SVM model which have the least impact on the ability to discriminate between the positive and negative examples. Shown are the ROC area under the curve values averaged from 10 independent feature elimination runs (Y axis) for each step in the process (X axis, showing the number of remaining features in the models). Error bars indicate +/−1 standard error. A significant drop in performance is observed when the number of features drops below 88. The identities of the conserved minimal feature set are shown in Figure 3 and implications discussed in the main text.
(0.15 MB TIF)

Table S1 List of organisms used in the phylogenetic profile input to SIEVE.
(0.04 MB XLS)

Table S2 Additional high-confidence Predictions in *S.* Typhimurium.
(0.04 MB XLS)

Table S3 Known or predicted secreted proteins in *C. trachomatis* not highly predicted by SIEVE.
(0.04 MB XLS)

Table S4 Unaligned N-terminal Sequences of Secreted Effectors and Agreement with SIEVE Positional Biases.
(0.04 MB XLS)

Table S5 Complete geneome SIEVE predictions for *S.* Typhimurium, *P. syringae*, *C. trachomatis*, *S. flexneri*, *V. cholerae*, and *Y. pestis*.
(4.15 MB XLS)

Text S1 Supplemental Methods and Information. In this section we present a complete and detailed description of the computational methods underlying the analysis described in the main text. We also include a number of results that support the main analysis including optimization of parameters for SIEVE, determination of confidence bounds for SIEVE predictions and several peripheral experiments designed to address the hypotheses presented in the main text.
(0.40 MB DOC)
