# Supplementary figures and images for: Accurate Prediction of Secreted Substrates and Identification of a Conserved Putative Secretion Signal for Type III Secretion Systems
Source: PLoS Pathog. 2009 Apr 24;5(4):e1000375. doi: 10.1371/journal.ppat.1000375 (PMC2668754; doi:10.1371/journal.ppat.1000375)

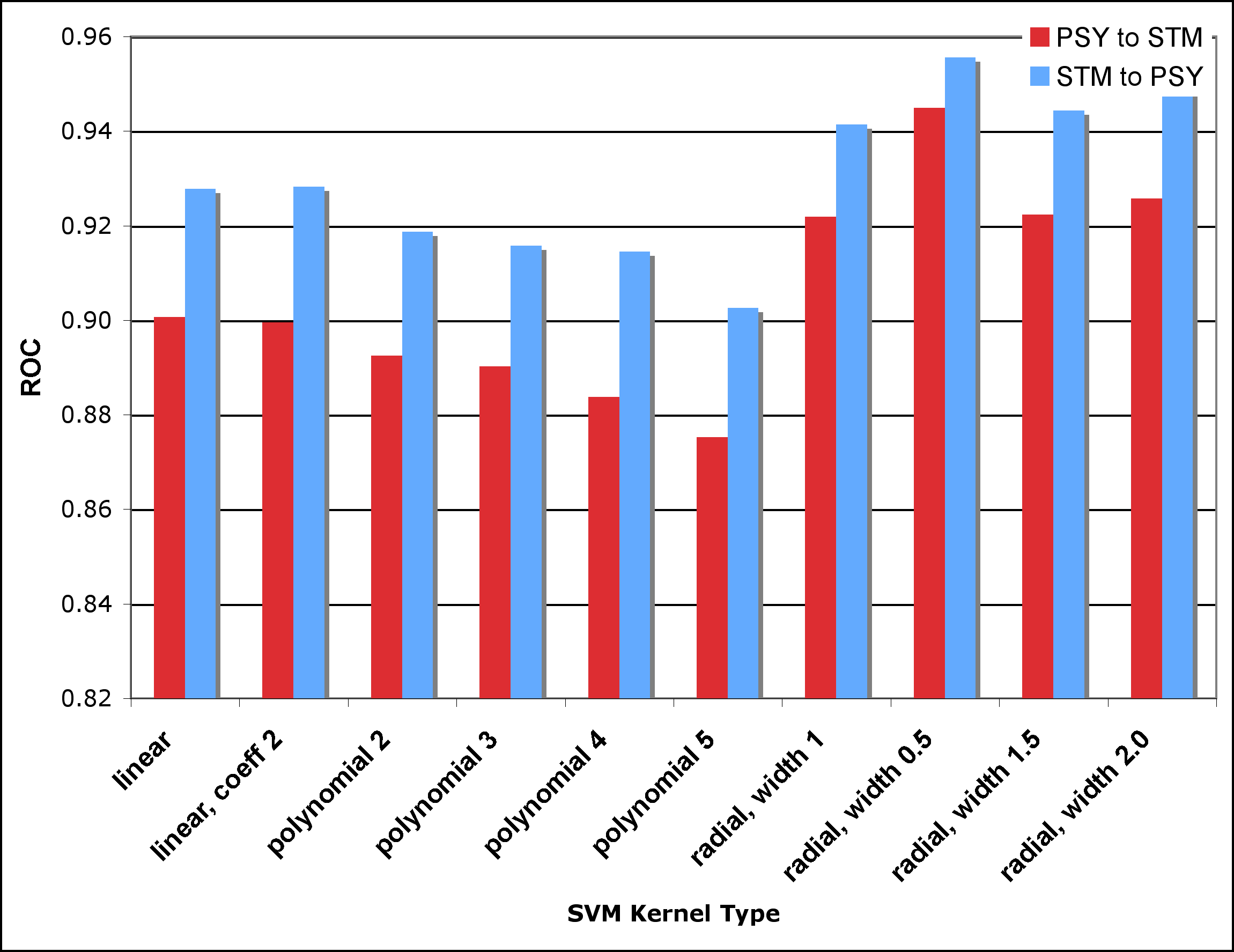

Supplement: Figure S1 — Performance of SIEVE models using different SVM kernel functions and parameters. The performance of the PSY to STM (red) and STM to PSY (blue) models were evaluated using the ROC area under the curve metric described in the text (Y axis). Performance using the radial basis function (radial) with a width parameter of 0.5 provided the best results for both models. (0.20 MB TIF) [file ppat.1000375.s001.tif]

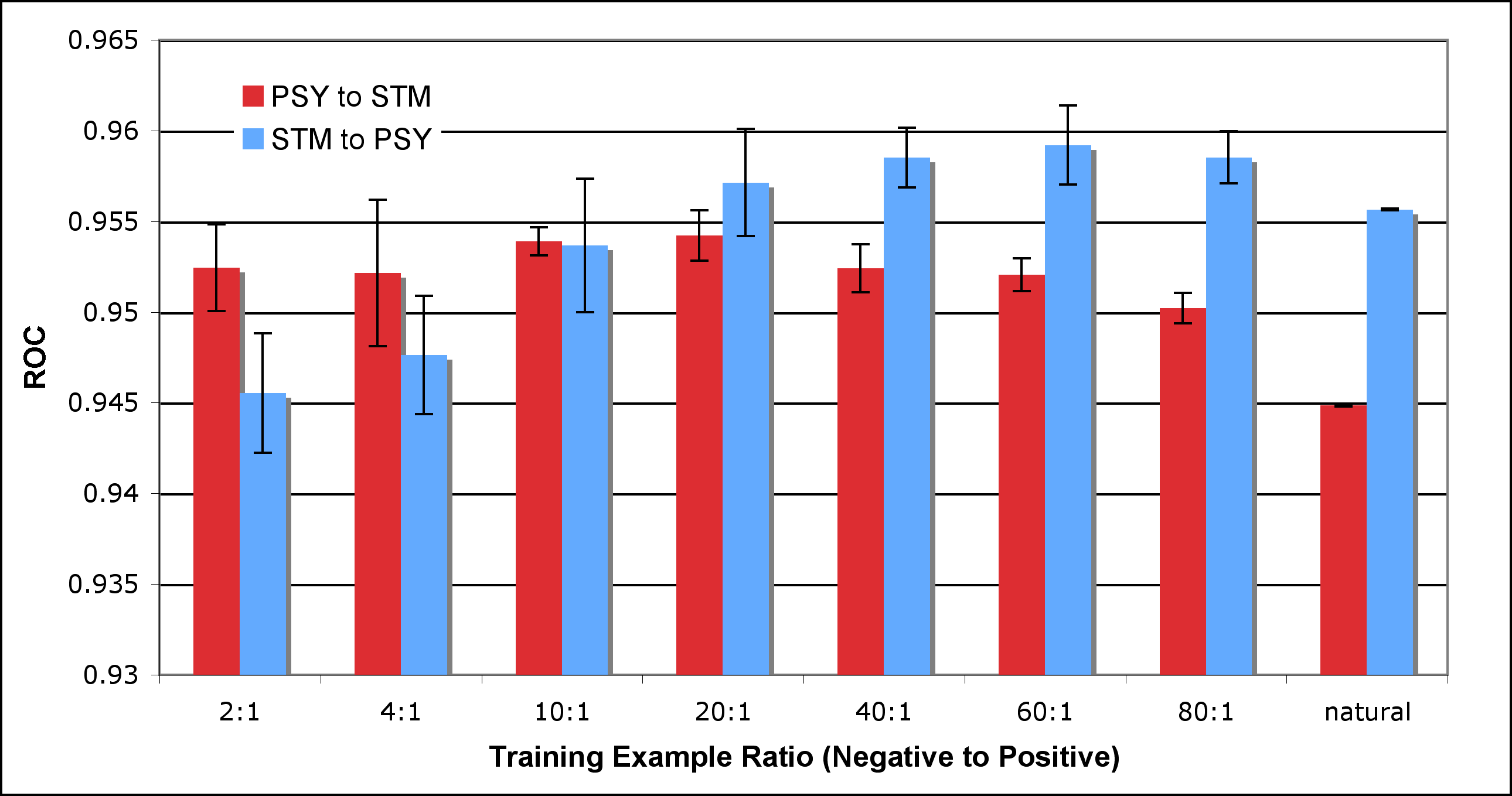

Supplement: Figure S2 — SIEVE performance using different ratios of negative top positive examples in the training process. The performance of the PSY to STM (red) and STM to PSY (blue) models were evaluated using the ROC area under the curve metric described in the text (Y axis). Models were trained with the radial basis function kernel and a width of 0.5 (see Figure S1) using the indicated ratio of negative to positive examples (X axis) and tested on the complete testing set (i.e. the entire set of positive and negative examples from the other organism). ‘Natural’ indicates that the entire set of negatives (all the proteins in the organism) were used for training. Error bars indicate +/−1 standard error calculated from 10 training runs using random selections of negative examples. The best performance is obtained using ratios of 20∶1 and 60∶1 for the STM to PSY and PSY to STM models, respectively. For consistency the ratio of 20∶1 was chosen for further SIEVE training in both models since it gives the best performance for the PSY to STM model and reasonable performance for the STM to PSY model. (0.17 MB TIF) [file ppat.1000375.s002.tif]

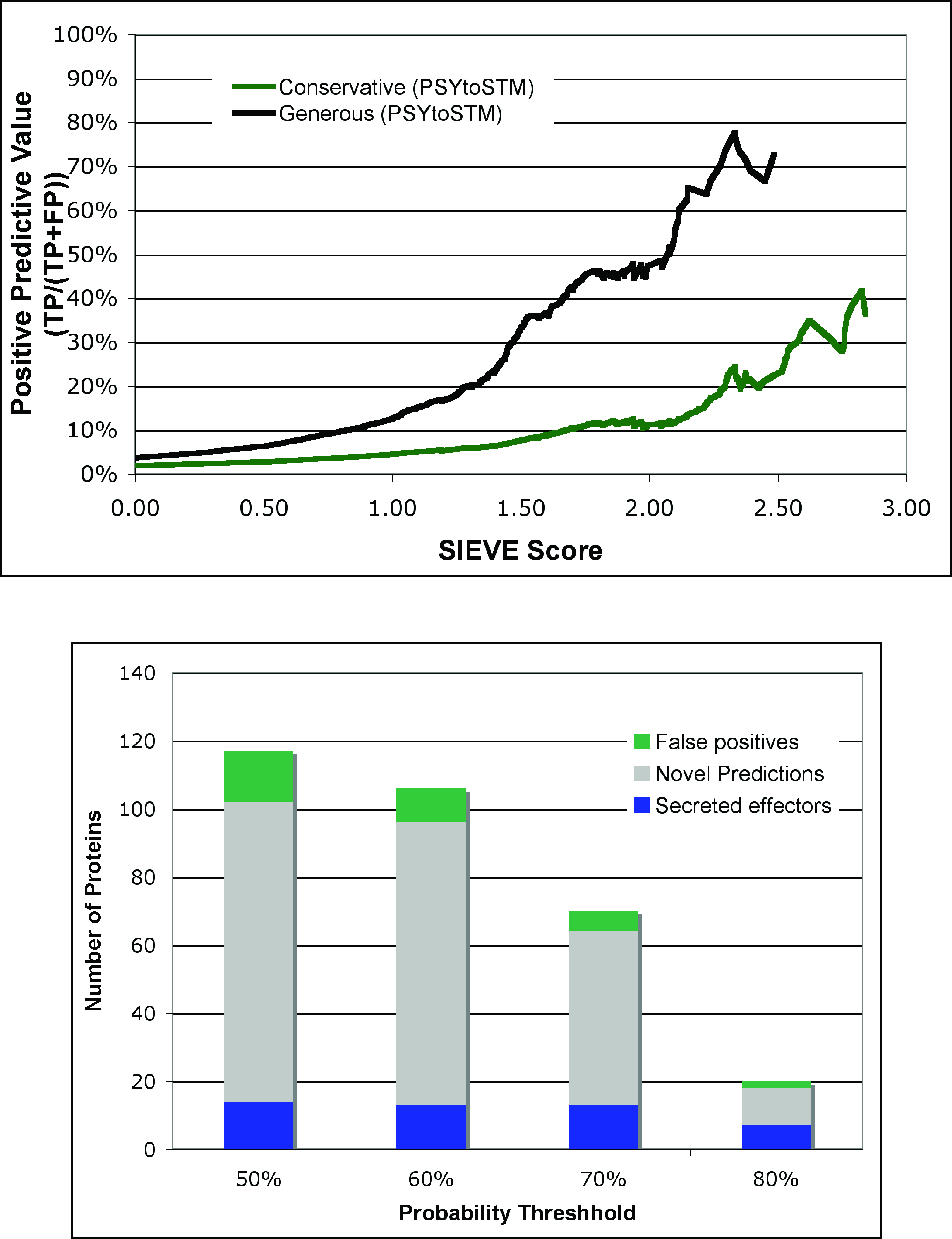

Supplement: Figure S3 — Estimation of SIEVE Prediction Confidence. A. The positive predictive value, the number of true positive predictions divided by the total number of predictions made at a particular score threshold (TP/(TP+FP)) is shown (Y axis) plotted against the SIEVE score threshold (X axis) for the conservative evaluation set, all proteins not experimentally determined to be secreted effectors are treated as negatives (green line), and the generous evaluation set, only proteins with known functions are considered as negative examples (black line). B. The number of false positive predictions (green; with known functions), novel predictions (grey; with no known function) and known secreted effectors (blue) are shown at several different confidence thresholds (as determined from the generous evaluation set). (3.00 MB TIF) [file ppat.1000375.s003.tif]

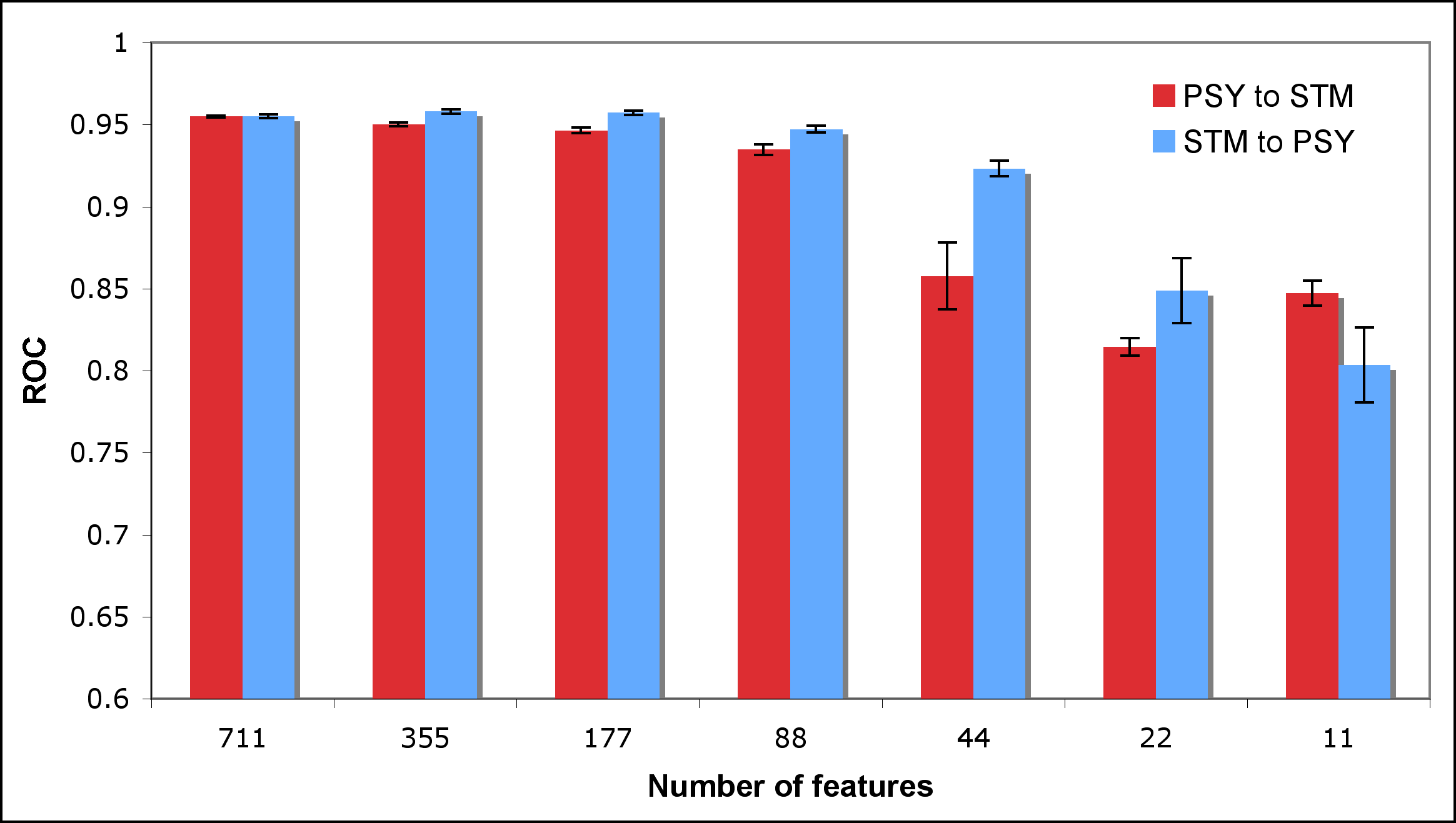

Supplement: Figure S4 — A minimal set of sequence-based features for accurate discrimination of type III secreted effectors. A recursive feature elimination approach was used that successively eliminates the 50% of the features in the SVM model which have the least impact on the ability to discriminate between the positive and negative examples. Shown are the ROC area under the curve values averaged from 10 independent feature elimination runs (Y axis) for each step in the process (X axis, showing the number of remaining features in the models). Error bars indicate +/−1 standard error. A significant drop in performance is observed when the number of features drops below 88. The identities of the conserved minimal feature set are shown in Figure 3 and implications discussed in the main text. (0.15 MB TIF) [file ppat.1000375.s004.tif]
