# Supplementary material for: Accurate Prediction of Secreted Substrates and Identification of a Conserved Putative Secretion Signal for Type III Secretion Systems
Source: PLoS Pathog. 2009 Apr 24;5(4):e1000375. doi: 10.1371/journal.ppat.1000375 (PMC2668754; doi:10.1371/journal.ppat.1000375)
Supplement: Text S1 — Supplemental Methods and Information. In this section we present a complete and detailed description of the computational methods underlying the analysis described in the main text. We also include a number of results that support the main analysis including optimization of parameters for SIEVE, determination of confidence bounds for SIEVE predictions and several peripheral experiments designed to address the hypotheses presented in the main text. (0.40 MB DOC) [file ppat.1000375.s010.doc]

**Text S1**

**Supplemental Methods and Information**

**Introduction**

In this section we present a complete and detailed description of the computational methods underlying the analysis described in the main text. We also include a number of results that support the main analysis including optimization of parameters for SIEVE, determination of confidence bounds for SIEVE predictions and several peripheral experiments designed to address the hypotheses presented in the main text.

**Methods**

***BLAST detection of secreted effectors in S. Typhimurium***

To provide a basis for comparison with our method, we obtained the set of known effectors used in [1]. We filtered out all the effectors that had been identified by sequence similarity alone (and not experimentally validated) and the known effectors from *S.* Typhimurium*,* leaving 182 known effectors from 29 species and/or strains. We then used BLAST to determine sequence similarity between this set and *S.* Typhimurium proteins.

***SVM methodology***

Support vector machines (SVM) are a class of machine learning methods which allow robust linear and non-linear classification of input vectors in an optimal fashion [2,3]. An SVM represents each example as a point in an N-dimensional space, where N is the number of numeric features chosen to represent the data. The sequence of numeric features that defines the N-dimensional point is referred to as a feature vector. The SVM then uses a function, called a kernel, to map the feature vector into an even higher-dimensional space that is linearly separable. This approach provides the optimal separation of the two classes given the chosen kernel and is resistant to overfitting problems encountered in other machine learning approaches. Solving the optimization problem to find the hyperplane is a quadratic programming problem, however the classification of a new input vector x is a linear computation:


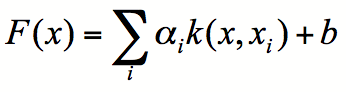


where (,b) defines the hyperplane and K(x,x_i_) is the kernel function (see below). For an overview of the SVM method see [2].

The kernel function is used during SVM training and classification tasks to compare input feature vectors with the support vectors, those vectors which are closest to the hyperplane. The kernel functions used in this study are: the linear kernel, which is the dot product of two vectors:


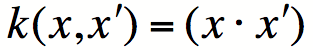


the non-linear polynomial kernel:


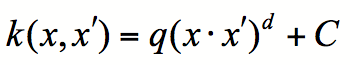


where d is the polynomial factor, *q* is a coefficient and C is a constant; and the non-linear radial basis function kernel:


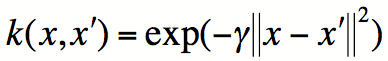


where γ is the width parameter. The non-linear kernels allow the SVM to map a non-linear classification problem into a higher dimensional space where a linear hyperplane can be found which separates the positive examples from the negative examples. Different kernels will provide different performance on a given problem and must be heuristically determined for a particular problem (see Figure S1).

We used the SVM software suite Gist [4] to perform all training, testing and evaluation of different models. Except where noted (e.g. Figure S1), we used a radial basis function kernel with a width of 0.5 for SIEVE classification.

***Positive and negative examples used in training and testing.*** An SVM is trained on a set of positive and negative examples, in this case known secreted effectors and proteins that have not been identified as effectors i.e. the remainder of the proteins in the organism, using a set of characteristics, or features, that have been derived from sequence analysis of the protein. The true set of negative examples is actually unknown; it is likely that a number of the proteins in our negative example set are secreted but have not been discovered yet. There are approaches to select a set of truly non-secreted proteins such as by using all proteins that are known to have well-defined roles in metabolic pathways or functional complexes (ribosomal proteins, e.g.). However, using a data integration approach like ours, narrowing the list of negative examples could easily make the classification task trivial. For example, proteins with well-defined metabolic functions are more likely to be conserved than secreted effectors on average, allowing very accurate discrimination based solely on sequence conservation. Even if the biases were not as directly evident, the performance would still be suspect. Therefore, with the exception of Figure S3, we chose to use the conservative set of all proteins that are not known to be secreted effectors as our negative example set for our analyses. This ensures that the performance we report using SIEVE is a conservative, lower-bound estimate, since it contains an unknown number of misclassified false-positive predictions (i.e. real secreted effectors that have not yet been discovered). To provide a generous (though not necessarily upper-bound) estimate of SIEVE performance we also use a more limited set of negative examples selected by including proteins with informative functional annotations (see Figure S3).

***Sequence-derived features***

Features are the different characteristics used as input to the SVM. The SVM uses the features to learn the difference between the positive examples, known secreted effectors in this case, and negative examples, proteins that have not been shown to be secreted effectors (see above). Features are numerically encoded as vectors, a sequence of numbers that can be thought of as a point in an N-dimensional space, where N is the number of features considered. SIEVE feature vectors represent a 711-dimensional space (see below).

Five sets of features were chosen for SIEVE based on their known or suspected distributions in secreted effectors:

***Sequence conservation (CON).*** We wanted to include a measure of sequence conservation that could be derived from BLAST output but that would provide a general measure of how conserved the sequence is based on alignment with other known sequences. Therefore we summarized conservation by two values representing the general evolutionary conservation of the entire protein sequence. The protein sequence was compared with all sequences in the NCBI non-redundant database using PSI-BLAST [5] and used to assemble a position-specific scoring matrix (PSSM) using all matched sequences with percentage sequence identity (PID) of greater than 20%. The PID measure is calculated as the ratio of the number of identical residues in the aligned region to the length of the query sequence. A PSSM represents each position in sequence alignment as the probability of observing each amino acid type in the alignment at that position. The PSSM was then used to calculate the information entropy at each sequence position. Information entropy is a measure of how much information is necessary to encode the distribution of amino acid probabilities: a lower entropy measure indicates that the position is more conserved since it takes less information to represent it. Entropy was calculated using Shannon’s uncertainty formula [6]:


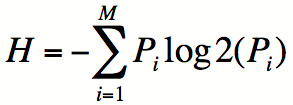


where M is the number of possible amino acids (20), Pi is the probability of observing a particular amino acid at position i, and H is in bits of information. The final measure of entropy was an average of the entropy at each sequence position. Both the average entropy measure for the entire PSSM in bits and the average entropy scaled to 0-1 were used as a two-dimensional feature vector for SIEVE.

***Phylogenetic profile (PHYL).*** Phylogenetic profiles are lists of significant sequence similarity (homology) found to proteins in a series of other organisms for each protein. They have been used to provide functional annotations and to predict interactions between proteins [7,8]. Phylogenetic profiles were constructed by determining the most similar protein in each of 54 organisms from all kingdoms (see Table S1). For each of the 54 organisms the percentage sequence identity (PID) and BLAST expectation value (evalue) were used as features. Representative organisms from the vertebrates were included in the profiles since a number of effectors have been shown to mimic host proteins functionally and some of these can be detected by sequence similarity [9]. Overall the phylogenetic profile provides information about the distribution of the protein over a range of different organisms with different evolutionary histories. For cases in which there was no detectable sequence similarity a value of 0 for the PID and a value of 10 was used for the evalue feature. The phylogenetic profiles from the 54 organisms were encoded as an 108-dimensional feature vector in SIEVE.

***Nucleotide composition (GC).*** The percentage of guanine and cytosine bases from the protein’s entire cognate nucleotide sequence was used [10]. This value was expressed as a zscore (i.e. the number of standard deviations from the mean) relative to the mean of the GC content from the set of all coding sequences in the genome, and used as a one-dimensional feature vector in SIEVE.

***Amino acid composition (AA).*** The amino acid composition was represented as the percentage representation for each of the possible 20 amino acids in the N-terminal 30 residues of the protein sequence [11-14]. This was used as a 20-dimensional feature vector in SIEVE.

***Sequence (SEQ).*** The N-terminal 30 residues [13,15], excluding the N-terminal methionine, were encoded as a vector by including 20 values for each sequence position corresponding to the 20 possible residue types. A value of 1 was assigned to the appropriate residue type and 0 to all others for each position. The importance of the N-terminal 30 residues is demonstrated in Results (Figure 2). No sequence alignment was performed. The positive example sets used for SIEVE were filtered to remove sequence similarity that is detectable by BLAST, and this means that ‘trivial’ similarities will not be detected by similarities in these vectors. This was used as a 580-dimensional feature vector in SIEVE.

All the features are based on data from the Bioverse database [16].

***Feature integration.*** Two methods are commonly used to integrate data of different kinds for classification by SVM [2]: kernel integration, which uses a different SVM kernel function for each kind of data, and feature integration, which combines different feature types into one single N-dimensional vector. We chose to use the feature integration approach and combined the feature sets listed below into a single vector with 711 dimensions, in which each dimension corresponds to a numeric value (described above).

***Training of the Intra-organism SIEVE Model***

For the model trained and tested on *S.* Typhimurium effectors (STM to STM), we wanted to evaluate all known effectors in the method so could not eliminate any from consideration completely. Instead we ensured that groups of similar effectors were treated as a unit; that is, a group of similar effectors was either in the training set or the testing set, but never in both, for purposes of evaluation. This was accomplished by grouping all 36 effectors into 27 groups based on sequence similarity detected by BLAST. This gave the following groups of similar effectors: SlrP, SseI, SseJ, SifA, SifB, and SspH-2; SopD-1 and SopD-2; GtgA and GogA; SseK-1 and SseK-2; PipB-1 and PipB-2; and 22 other effectors unique in *S.* Typhimurium. These groups were kept together for the purposes of training, testing and performance evaluation. For example, if PipB-1 was included in the training set, PipB-2 was also included to avoid identification of the homolog. This process ensures that the performance of the method was not affected by the similarity between sequences detectable by BLAST.

***Determination of most important input features.*** Recursive feature elimination (RFE) is a process to determine which input features are the most important in the classification task. It is performed by repeatedly training and testing an SVM model on a set of examples, successively eliminating the features with the lowest impact on the performance of the model. We performed feature elimination using the gist-rfe program, part of the Gist suite, with the parameters indicated in results. Since RFE uses a random process to initially eliminate features it gives somewhat different results each time it is performed using the same examples. We therefore repeated the feature elimination 10 times using random sets of negative examples for training and testing. Final significant features were limited to those that were shared in 8 or more of the different feature elimination runs.

***Leave-one-out evaluation of same-organism predictions.*** It is impossible to reliably assess the performance of a trained model using only the examples on which it was trained. Therefore, to assess the performance of SIEVE when trained and tested on the same set of effectors (the STM to STM model described below) we used a leave-one-out approach in which each unique group of secreted effectors (see above) was tested against a model trained on all the other positive examples (not including the group being tested). As determined by optimizing parameters (below) negative examples were included in the training at a ratio of 20:1 and the radial basis function kernel with a width of 0.5 was used for SVM classification. SIEVE was trained on 26 of the groups of positive effectors with approximately 700 randomly selected negative examples (1:20), then tested on the positive group that was left out with a ‘natural’ ratio of negatives 1:135. Training and testing was repeated 10 times for each effector group using randomly selected sets of negative examples to include in the training and testing examples. Each protein is tested multiple times (approximately 10 times for each negative example) and scores calculated as the average SVM discriminant for that protein. Final SIEVE scores are expressed as the zscore (number of standard deviations from the mean) of the SVM discriminant score for each protein. The mean of SVM discriminant scores for the STM to STM model is -0.88 with a standard deviation of 0.2, therefore an SVM discriminant value of 0.2 will have a zscore of 5.4.

**Results**

***Optimization of SIEVE.*** The kernel function is used to map feature vectors from a non-linear classification problem (defined by the positive and negative examples and their associated feature vectors; see Methods) into a higher dimensional space to allow the determination of a linear hyperplane that provides the best classification. We tested several different kernel functions in the SVM to see which would provide the best performance on the dataset for our problem. We tested the linear kernel and two non-linear kernels; the polynomial kernel with several exponents and the radial basis function kernel with several widths (see Methods). We evaluated the SIEVE model trained on *P. syringae* proteins and tested on *S.* Typhimurium proteins (PSY to STM) and the reverse experiment of SIEVE trained on *S.* Typhimurium proteins and tested on *P. syringae* proteins (STM to PSY). The results and performance of both these models are discussed in the main text. As evaluated by the area under the ROC curve the radial basis function kernel performed better than the others tested (Figure S1).

The performance of an SVM classifier is highly dependent on the selection of examples used for training. False positive predictions are positive predictions that are not truly secreted effectors and false negative predictions are true secreted effectors that are not predicted by the method. If a small number of negative examples are used in the training process the method may not generalize well to new examples and thus give a high false-positive rate. Conversely, since an assumption of this work is that there are an unknown number of novel secreted effectors in the set used for negative examples, including too many of these might cause the method to produce more false-negative predictions. We therefore tested the number of negative examples to include in the training set to maximize performance. We used the same set of positive examples but varied the ratio of the negative examples to positive examples used during the training process from 2:1 to the naturally occurring ratio for that organism (i.e. for *P. syringae,* 29 positive examples and ~5100 negative examples). These models were then tested on the naturally occurring examples from the opposite organism. We found that the 20:1 negative to positive ratio examples provided the best performance for the PSY to STM model and the 60:1 negative to positive ratio provided the best performance for the STM to PSY model (Figure S2). To establish a general strategy for SIEVE we chose to use the 20:1 ratio since it provided the best performance considering both models.

***Inter-organism Prediction of Secreted Effectors.*** The resulting best SIEVE models used the radial basis function with a width parameter of 0.5, the 20:1 negative to positive example ratios and a sequence length of 30 residues. This gives very good classification with ROC values of 0.95 and 0.96 for the PSY to STM and STM to PSY models, respectively. By contrast, partial least squares regression discriminant analysis [17], applied to the same positive and negative examples and feature vectors gave AUC values of 0.76 and 0.82, respectively. Partial least squares regression is a statistical method for classification which finds a linear statistical model to make predictions based on observed variables, in this case the feature vectors used in SIEVE. This shows that using a non-linear SVM provides a significant improvement in performance over linear classification methods for this problem.

**Intra-organism Prediction of Secreted Effectors**. We were also interested in assessing the performance of SIEVE when trained and tested on effectors from a single organism. We first sorted effectors in *S.* Typhimurium into groups on the basis of detectable sequence similarity (see Methods). We then applied a leave-one-out approach in which SIEVE was trained on 26 effector groups and then tested on the remaining effector group. This analysis (STM to STM model) gave an ROC of 0.96, apparently identical to the results from the PSY to STM model. However, as seen in Table 1, each model ranked the known secreted effectors differently suggesting that results could be improved by combining the two approaches. Indeed, using positive and negative examples from *P. syringae* in the SIEVE training then testing using the same leave-one-out approach yielded a modestly improved ROC AUC of 0.97. These results show that SIEVE can be used to accurately predict secreted effectors within and across organisms and that combining examples from different organisms can improve discrimination of secreted effectors.

**Classification of Effectors Using Sequence Alone.** From the analysis of sequence length (Figure 2A) it can be seen that, though the combination of features other than sequence can provide good discrimination (e.g. an ROC of ~0.87), the addition of the SEQ features provides a significant gain in performance for both models. We were interested in determining how well the SEQ features alone could perform and so we used the sequence information alone from the N-terminal 30 residues to classify secreted effectors. This analysis gives an ROC of 0.81 for the PSY to STM SEQ-only model and 0.88 for the STM to PSY SEQ-only model, nearly as good as the combination of other features by themselves. The PSY to STM model has a specificity of 83% at a sensitivity of 80% meaning that it is able to classify 83% (24) of the known effectors in the top 20% of predictions for the entire genome.

***Determination of a Minimal Feature Set for Classification.*** To determine the most important sequence-derived features for the classification task in each of the models we used a recursive elimination approach. The model is first trained as described using all the initial features from the five groups listed above; conservation (CONS), phylogenetic profile (PHYL), G+C content (GC), amino acid composition (AA), and sequence (SEQ). The model is then tested against a set of examples not used in the training and the features are ranked based on their importance in the classification task. The least important features are eliminated and the process is repeated using the remaining features. A minimal set of sequence-derived features necessary for accurate classification can be determined by evaluating the performance of the model at each successive step, which includes fewer and fewer features. This minimal set can then be examined to draw biological inferences. The results of this process are shown in Figure S4. In both cases (STM to PSY and PSY to STM) models with fewer than 88 of the original 711 features show a significant drop in performance, therefore we examined the remaining 88 features for both models (Figure 3) to determine the most important biases in the models . Because each recursive feature elimination run can yield different results we considered features that were retained by 8 or more of 10 feature elimination runs as a minimal conserved feature set for each model.

Examining the remaining minimal feature sets for both organisms showed that the evolutionary conservation measures (CONS) were important for discrimination of effectors in both *S.* Typhimurium and *P. syringae*. The importance of the conservation measure is likely due to the lack of conservation observed in secreted effectors. The G+C content (GC) was more important in the discrimination of *P. syringae* effectors, possibly because *P. syringae* has a lower overall G+C content (41.8% in coding regions) than *S.* Typhimurium (52.6%). Presumably, the G+C content of *P. syringae* effectors (45.8%) allows better discrimination than the G+C content of *S.* Typhimurium effectors (42.6%), though both are significantly different from their backgrounds. Finally, both minimal feature sets incorporated features from the phylogenetic profiles, most notably that similarity to proteins in species closely related to the training species (*P. aeruginosa* for *P. syringae* and *E. coli* for *S.* Typhimurium) was a negative predictor of effector classification in the target species. This result may indicate that SIEVE is filtering out false positive predictions that arise from components of the type III secretion system apparatus or related complexes such as the flagella, which are more conserved than secreted effectors.

A full discussion of the identified sequence motifs identified is presented in the main text.

***Estimation of Confidence Range for SIEVE Predictions.*** The probability of a SIEVE prediction being a true secreted effector can be estimated using precision (TP/(TP+FP)). The validity of this estimate is highly dependent upon the completeness of the set of true positive and true negative examples used for evaluation. As discussed previously it is our hypothesis that there are an undetermined number of true secreted effectors that have not yet been experimentally characterized and are therefore misclassified true negative examples. This hypothesis is supported by the findings presented that three highly scored predictions have been shown to be novel type III secreted effectors in *S.* Typhimurium (see main text). Therefore probabilities estimated using the entire set of examples represent the lower bounds, the most conservative estimates for the probability of being a secreted effector.

To provide a set of more generous probability estimates we re-evaluated the predictions made by the PSY to STM model. Genes with characterized functions are unlikely to be secreted effectors therefore we first eliminated from consideration all genes without a gene name then removed genes with uninformative annotations, e.g. those labeled as “putative” or “hypothetical”. These simple filters provide a generous set for evaluation in that only those genes which have defined functions will be counted as negative examples. Finally, we reclassified the three novel secreted effectors described in the main text (spvC, spvD and pagD) as true positives. The filtered examples gave an ROC of 0.985. The positive predictive values (Y axis) of the conservative and generous evaluation sets are shown in Figure S3A as a function of the SIEVE score (X axis). Figure S3B shows the number of false-positive predictions (i.e. those with defined function), novel predictions (i.e. those with no defined function), and secreted effectors at several probability thresholds. These evaluation sets give conservative and generous bounds for the probability of a prediction being a true secreted effector. Even in the generous evaluation set, only one (invE) of the five highest scoring false positive predictions (in order; invE, ssaP, traX, spvA, and psiA) has been experimentally demonstrated not to be secreted [18]. This highlights the difficulty in determining a set of true negative examples. Further experimental validation of these predictions will provide a better measure of confidence for SIEVE predictions.

**Figure Legends**

**Figure S1. Performance of SIEVE models using different SVM kernel functions and parameters.** The performance of the PSY to STM (red) and STM to PSY (blue) models were evaluated using the ROC area under the curve metric described in the text (Y axis). Performance using the radial basis function (radial) with a width parameter of 0.5 provided the best results for both models.


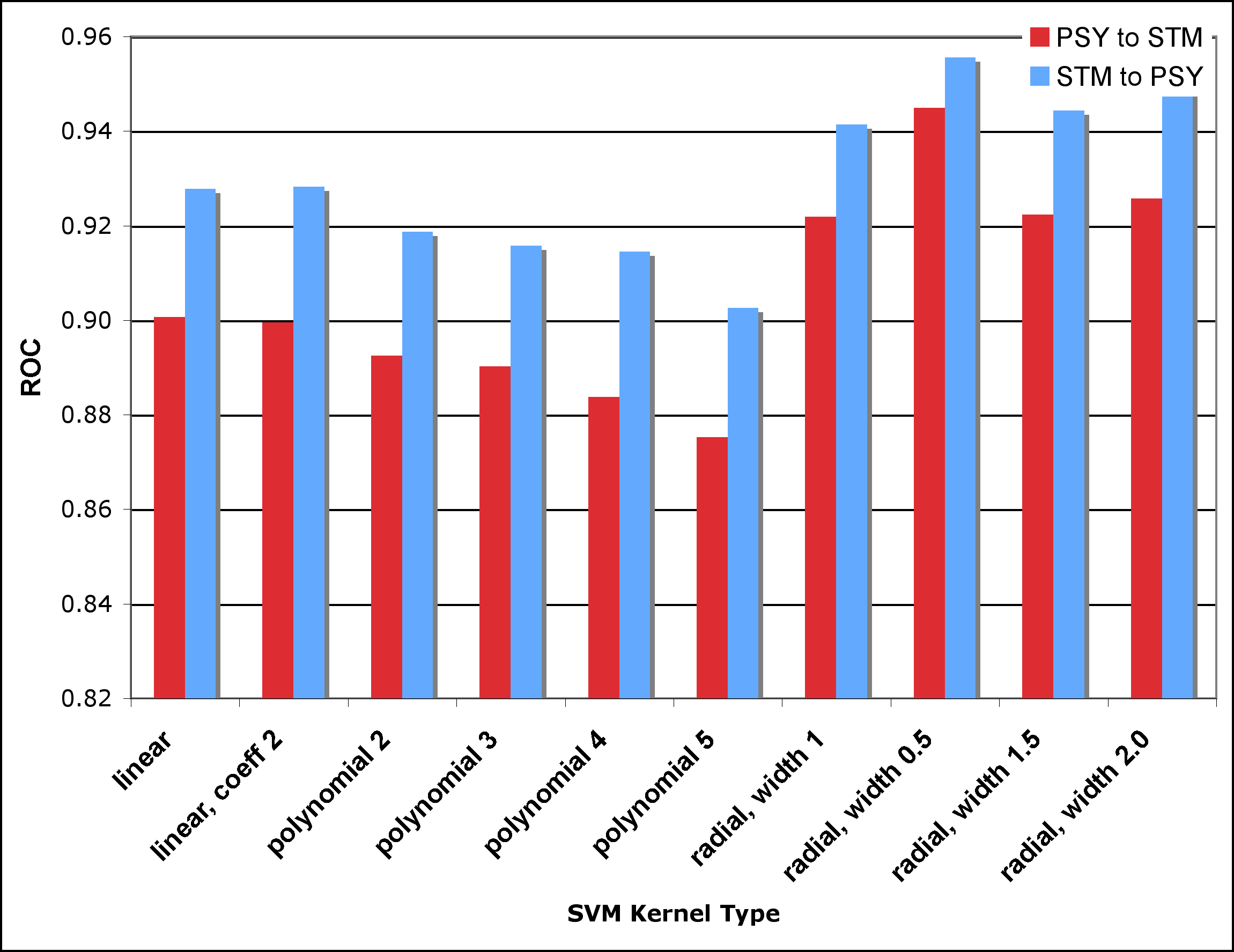


**Figure S2. SIEVE performance using different ratios of negative to positive examples in the training process.** The performance of the PSY to STM (red) and STM to PSY (blue) models were evaluated using the ROC area under the curve metric described in the text (Y axis). Models were trained with the radial basis function kernel and a width of 0.5 (see Figure S1) using the indicated ratio of negative to positive examples (X axis) and tested on the complete testing set (i.e. the entire set of positive and negative examples from the other organism). ‘Natural’ indicates that the entire set of negatives (all the proteins in the organism) were used for training. Error bars indicate +/- 1 standard error calculated from 10 training runs using random selections of negative examples. The best performance is obtained using ratios of 20:1 and 60:1 for the STM to PSY and PSY to STM models, respectively. For consistency the ratio of 20:1 was chosen for further SIEVE training in both models since it gives the best performance for the PSY to STM model and reasonable performance for the STM to PSY model.

**
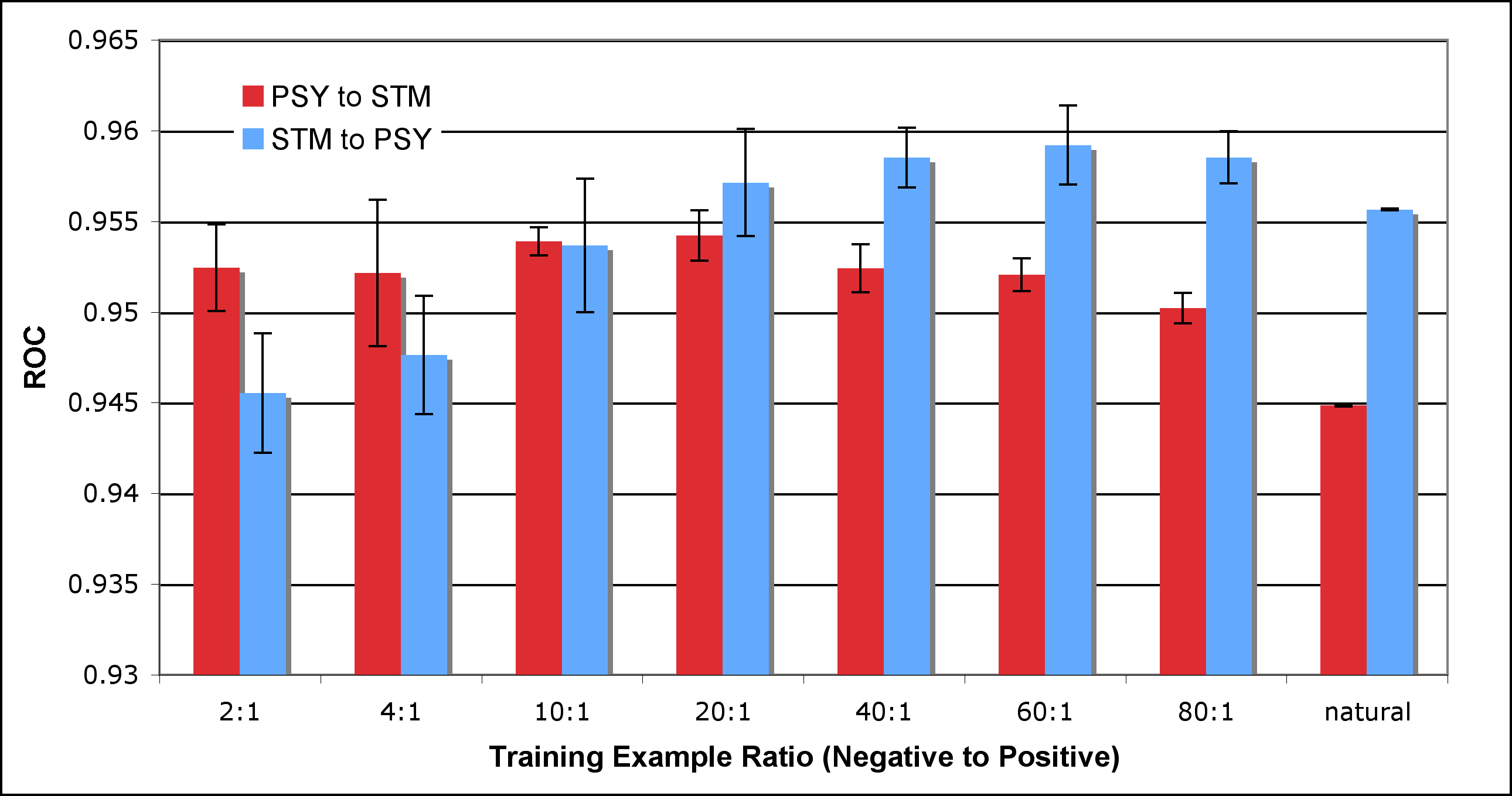
**

**Figure S3. Estimation of SIEVE Prediction Confidence. A.** The positive predictive value, the number of true positive predictions divided by the total number of predictions made at a particular score threshold (TP/(TP+FP)) is shown (Y axis) plotted against the SIEVE score threshold (X axis) for the conservative evaluation set, all proteins not experimentally determined to be secreted effectors are treated as negatives (green line), and the generous evaluation set, only proteins with known functions are considered as negative examples (black line). **B.** The number of false positive predictions (green; with known functions), novel predictions (grey; with no known function) and known secreted effectors (blue) are shown at several different confidence thresholds (as determined from the generous evaluation set).


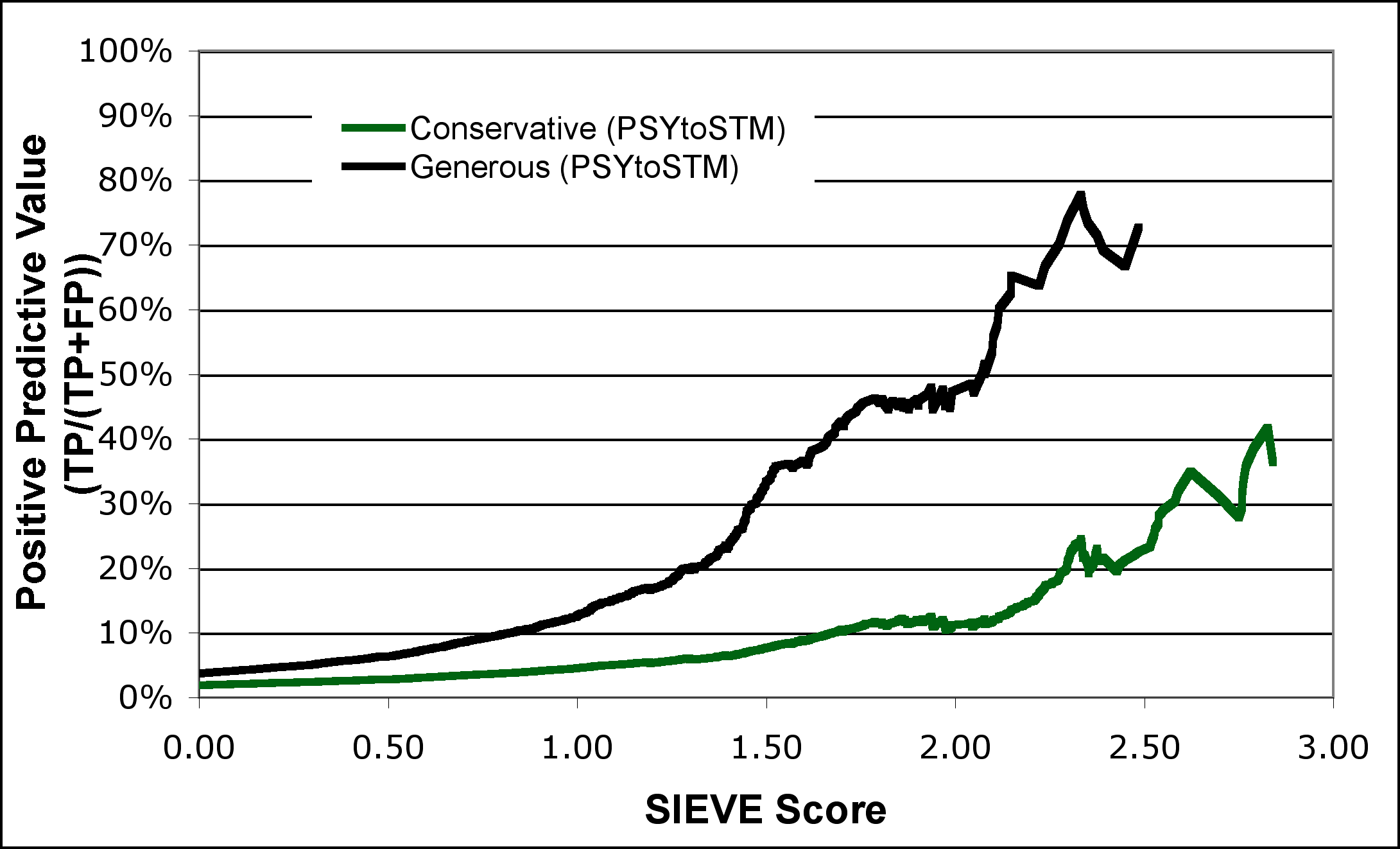


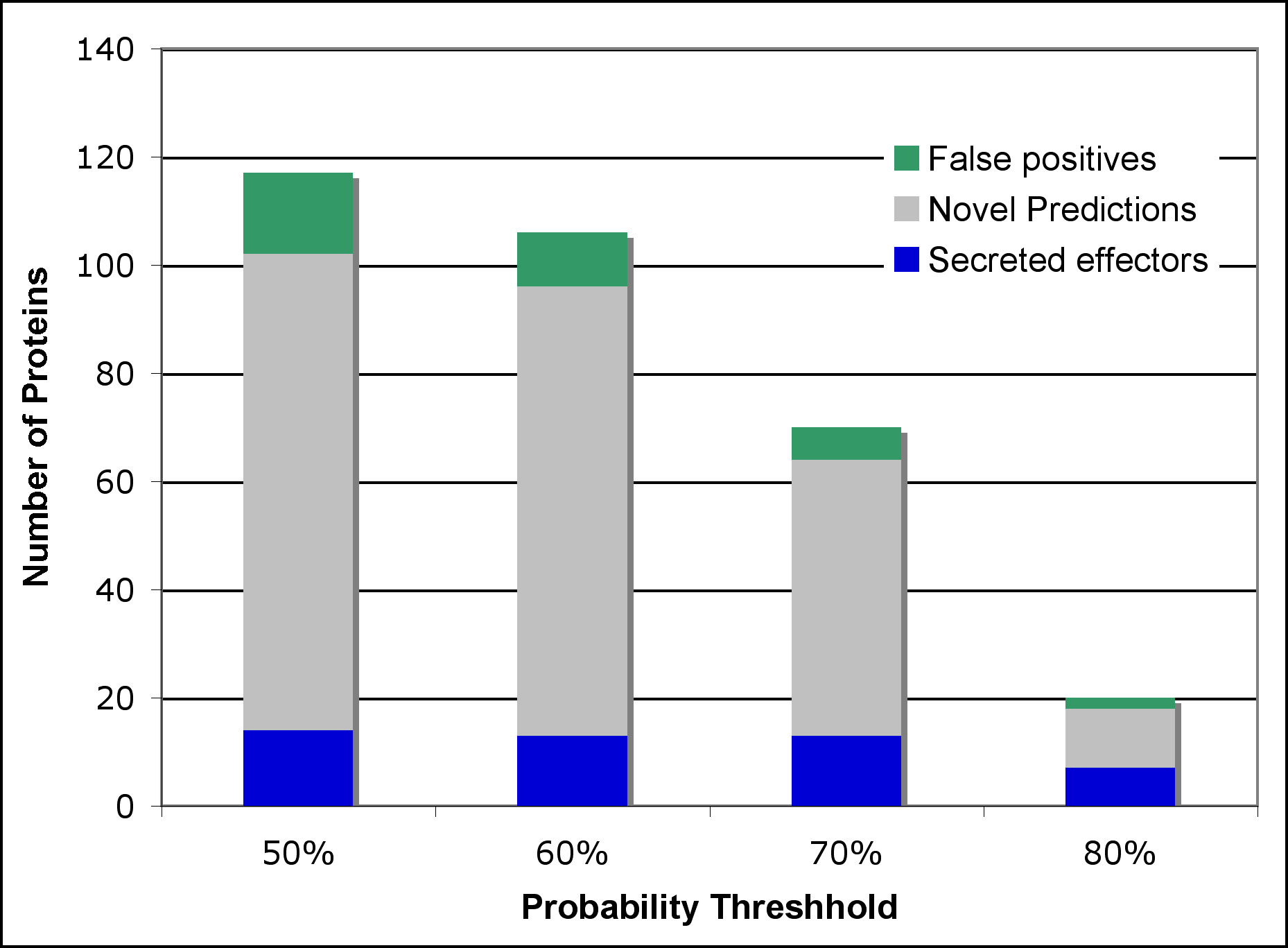


**Figure S4. A minimal set of sequence-based features for accurate discrimination of type III secreted effectors.** A recursive feature elimination approach was used that successively eliminates the 50% of the features in the SVM model which have the least impact on the ability to discriminate between the positive and negative examples. Shown are the ROC area under the curve values averaged from 10 independent feature elimination runs (Y axis) for each step in the process (X axis, showing the number of remaining features in the models). Error bars indicate +/- 1 standard error. A significant drop in performance is observed when the number of features drops below 88. The identities of the conserved minimal feature set are shown in Figure 3 and implications discussed in the main text.


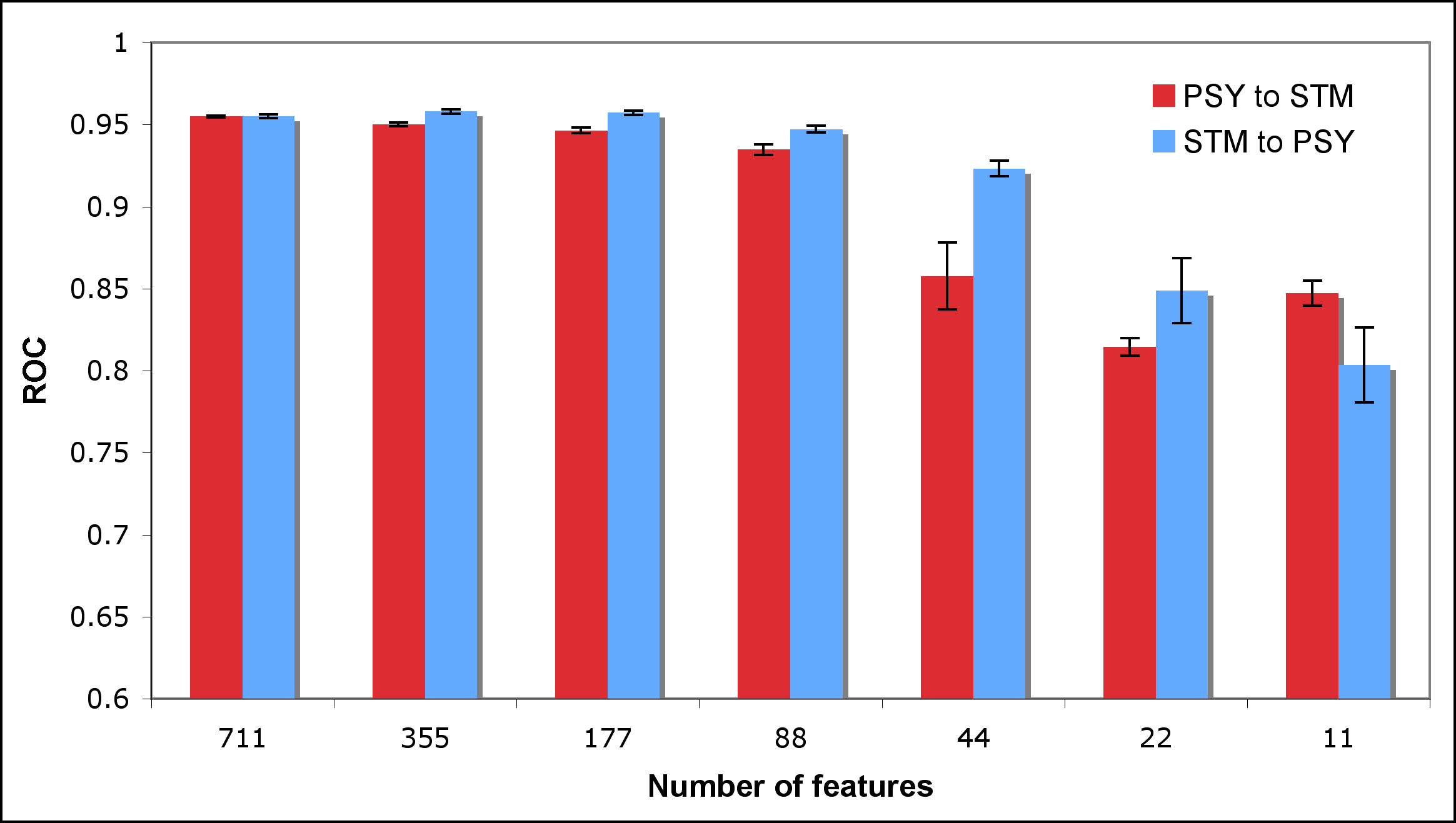


**References**

References

1. Tobe T, Beatson SA, Taniguchi H, Abe H, Bailey CM, et al. (2006) An extensive repertoire of type III secretion effectors in Escherichia coli O157 and the role of lambdoid phages in their dissemination. Proc Natl Acad Sci U S A 103: 14941-14946.

2. Noble WS (2006) What is a support vector machine? Nat Biotechnol 24: 1565-1567.

3. Vapnik W (1995) The Nature of Statistical Learning Theory. New York: Springer.

4. Pavlidis P, Wapinski I, Noble WS (2004) Support vector machine classification on the web. Bioinformatics 20: 586-587.

5. Altschul SF, Madden TL, Schaffer AA, Zhang J, Zhang Z, et al. (1997) Gapped BLAST and PSI-BLAST: a new generation of protein database search programs. Nucleic Acids Res 25: 3389-3402.

6. Shannon CE (1948) A Mathematical Theory of Communication. Bell Syst Tech J 27: 379-423, 623-656.

7. Date SV, Marcotte EM (2003) Discovery of uncharacterized cellular systems by genome-wide analysis of functional linkages. Nat Biotechnol 21: 1055-1062.

8. Pellegrini M, Marcotte EM, Thompson MJ, Eisenberg D, Yeates TO (1999) Assigning protein functions by comparative genome analysis: protein phylogenetic profiles. Proc Natl Acad Sci U S A 96: 4285-4288.

9. Desveaux D, Singer AU, Dangl JL (2006) Type III effector proteins: doppelgangers of bacterial virulence. Curr Opin Plant Biol 9: 376-382.

10. Rohmer L, Guttman DS, Dangl JL (2004) Diverse evolutionary mechanisms shape the type III effector virulence factor repertoire in the plant pathogen Pseudomonas syringae. Genetics 167: 1341-1360.

11. Lloyd SA, Forsberg A, Wolf-Watz H, Francis MS (2001) Targeting exported substrates to the Yersinia TTSS: different functions for different signals? Trends Microbiol 9: 367-371.

12. Lloyd SA, Sjostrom M, Andersson S, Wolf-Watz H (2002) Molecular characterization of type III secretion signals via analysis of synthetic N-terminal amino acid sequences. Mol Microbiol 43: 51-59.

13. Schechter LM, Vencato M, Jordan KL, Schneider SE, Schneider DJ, et al. (2006) Multiple approaches to a complete inventory of Pseudomonas syringae pv. tomato DC3000 type III secretion system effector proteins. Mol Plant Microbe Interact 19: 1180-1192.

14. Vinatzer BA, Jelenska J, Greenberg JT (2005) Bioinformatics correctly identifies many type III secretion substrates in the plant pathogen Pseudomonas syringae and the biocontrol isolate P. fluorescens SBW25. Mol Plant Microbe Interact 18: 877-888.

15. Petnicki-Ocwieja T, Schneider DJ, Tam VC, Chancey ST, Shan L, et al. (2002) Genomewide identification of proteins secreted by the Hrp type III protein secretion system of Pseudomonas syringae pv. tomato DC3000. Proc Natl Acad Sci U S A 99: 7652-7657.

16. McDermott J, Samudrala R (2003) Bioverse: functional, structural and contextual annotation of proteins and proteomes. Nucleic Acids Res 31: 3736-3737.

17. Boulesteix AL, Strimmer K (2007) Partial least squares: a versatile tool for the analysis of high-dimensional genomic data. Brief Bioinform 8: 32-44.

18. Kubori T, Galan JE (2002) Salmonella type III secretion-associated protein InvE controls translocation of effector proteins into host cells. J Bacteriol 184: 4699-4708.

19. Bannantine JP, Griffiths RS, Viratyosin W, Brown WJ, Rockey DD (2000) A secondary structure motif predictive of protein localization to the chlamydial inclusion membrane. Cell Microbiol 2: 35-47.

20. Li Z, Chen C, Chen D, Wu Y, Zhong Y, et al. (2008) Characterization of fifty putative inclusion membrane proteins encoded in the Chlamydia trachomatis genome. Infect Immun 76: 2746-2757.

21. Lugert R, Kuhns M, Polch T, Gross U (2004) Expression and localization of type III secretion-related proteins of Chlamydia pneumoniae. Med Microbiol Immunol 193: 163-171.

22. Subtil A, Delevoye C, Balana ME, Tastevin L, Perrinet S, et al. (2005) A directed screen for chlamydial proteins secreted by a type III mechanism identifies a translocated protein and numerous other new candidates. Mol Microbiol 56: 1636-1647.
